# NAT10-mediated upregulation of *GAS5* facilitates immune cell infiltration in non-small cell lung cancer via the MYBBP1A-p53/IRF1/type I interferon signaling axis

**DOI:** 10.1038/s41420-024-01997-2

**Published:** 2024-05-18

**Authors:** Zimu Wang, Jing Luo, Hairong Huang, Li Wang, Tangfeng Lv, Zhaofeng Wang, Chuling Li, Yimin Wang, Jiaxin Liu, Qinpei Cheng, Xueying Zuo, Liwen Hu, Mingxiang Ye, Hongbing Liu, Yong Song

**Affiliations:** 1 Department of Respiratory and Critical Care Medicine, Affiliated Jinling Hospital, Medical School of Nanjing University, Nanjing, 210002 China; 2 Department of Respiratory and Critical Care Medicine, Nanjing Drum Tower Hospital, Nanjing University School of Medicine, Nanjing, 210008 China; 3 Department of Cardiothoracic Surgery, Affiliated Jinling Hospital, Medical School of Nanjing University, Nanjing, 210002 China; 4 Nanjing Medical University, Nanjing, 211166 China; 5 Department of Respiratory and Critical Care Medicine, Affiliated Jinling Hospital, Nanjing Medical University, Nanjing, 210002 China

**Keywords:** Non-small-cell lung cancer, Cancer microenvironment

## Abstract

Interactions of tumor cells with immune cells in the tumor microenvironment play an important role during malignancy progression. We previously identified that *GAS5* inhibited tumor development by suppressing proliferation of tumor cells in non-small cell lung cancer (NSCLC). Herein, we discovered a tumor-suppressing role for tumor cell-derived *GAS5* in regulating tumor microenvironment. *GAS5* positively coordinated with the infiltration of macrophages and T cells in NSCLC clinically, and overexpression of *GAS5* promoted macrophages and T cells recruitment both in vitro and in vivo. Mechanistically, *GAS5* stabilized p53 by directly binding to MYBBP1A and facilitating MYBBP1A-p53 interaction, and enhanced p53-mediated transcription of IRF1, which activated type I interferon signaling and increased the production of downstream CXCL10 and CCL5. We also found that activation of type I interferon signaling was associated with better immunotherapy efficacy in NSCLC. Furthermore, the stability of *GAS5* was regulated by NAT10, the key enzyme responsible for N4-acetylcytidine (ac4C) modification, which bound to *GAS5* and mediated its ac4C modification. Collectively, tumor cell-derived *GAS5* could activate type I interferon signaling via the MYBBP1A-p53/IRF1 axis, promoting immune cell infiltration and potentially correlating with immunotherapy efficacy, which suppressed NSCLC progression. Our results suggested *GAS5* as a promising predictive marker and potential therapeutic target for combination therapy in NSCLC.

**Figure:**
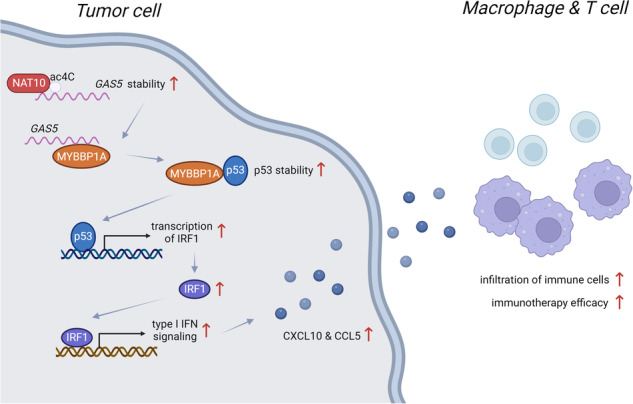
A schematic diagram demonstrating the regulatory effect of GAS5 on immune cell infiltration by activating type I interferon signaling via MYBBP1A-p53/IRF1 axis in non-small cell lung cancer. IFN, interferon.

A schematic diagram demonstrating the regulatory effect of GAS5 on immune cell infiltration by activating type I interferon signaling via MYBBP1A-p53/IRF1 axis in non-small cell lung cancer. IFN, interferon.

## Introduction

Lung cancer is the most common cause of cancer-related death and non-small cell lung cancer (NSCLC) accounts for the majority of lung cancer [1]. The initiation, growth, and metastasis of lung cancer are greatly influenced by the tumor microenvironment (TME), which is composed of tumor cells, infiltrating immune cells, cancer-associated fibroblasts, extracellular matrix, and other components [2]. Treatment strategy of lung cancer has been changed by immunotherapy in recent years, which targets TME and has improved survival of NSCLC patients [2]. Tumors exhibiting ‘hot’ immune signature are infiltrated with more immune cells and patients with these tumors are more likely to benefit from immunotherapy [3]. The TME can be modulated by tumor cells via the secretion of cytokines, chemokines, and exosomes [4], and a better understanding of this interaction is crucial for the development of novel diagnosis, prognosis, and treatment strategy of NSCLC.

Type I interferon signaling was initially discovered for its antiviral effects [5], and its role in tumor biology has been explored. Generally, activation of the type I interferon signaling exhibits anti-tumor effects, such as inhibiting the growth and metastasis of tumor cells and regulating the infiltration of immune cells [6]. These effects are achieved by inducing the expression of interferon-stimulated genes (ISGs), including genes encoding chemokines and major histocompatibility complex (MHC) molecules [7, 8]. Type I interferon signaling also mediates tumor response to immunotherapy [9, 10]. Novel anticancer therapeutic approaches involving type I interferon signaling are being explored, some of which are already in phases of clinical trials [6].

Long non-coding RNAs (lncRNAs) are RNAs that contain more than 200 nucleotides in length and cannot be translated into functional proteins [11]. LncRNA growth arrest-specific 5 (*GAS5*) was originally discovered in growth-arrested cells [12] and has been reported to be involved in multiple diseases by regulating NF-κB pathway [13], cAMP/CREB pathway [14], Wnt/β-catenin pathway [15] and etc. We previously found that *GAS5* was downregulated and associated with worse clinicopathological characteristics in NSCLC, and suppressed growth and migration of tumor cells by increasing p53 expression at post-transcription level [16]. In the current study, we further explored the influence of tumor cell-derived *GAS5* on immune cells in the TME and the underlying mechanism. We found that *GAS5* modulated the infiltration of macrophages and T cells by activating the type I interferon signaling via MYBBP1A-p53/IRF1 axis in NSCLC, which might enhance immunotherapy efficacy, indicating a promising predictive and therapeutic target for NSCLC.

## Results

### *GAS5* inhibits NSCLC development and promotes immune cell infiltration

**Fig. 1:**
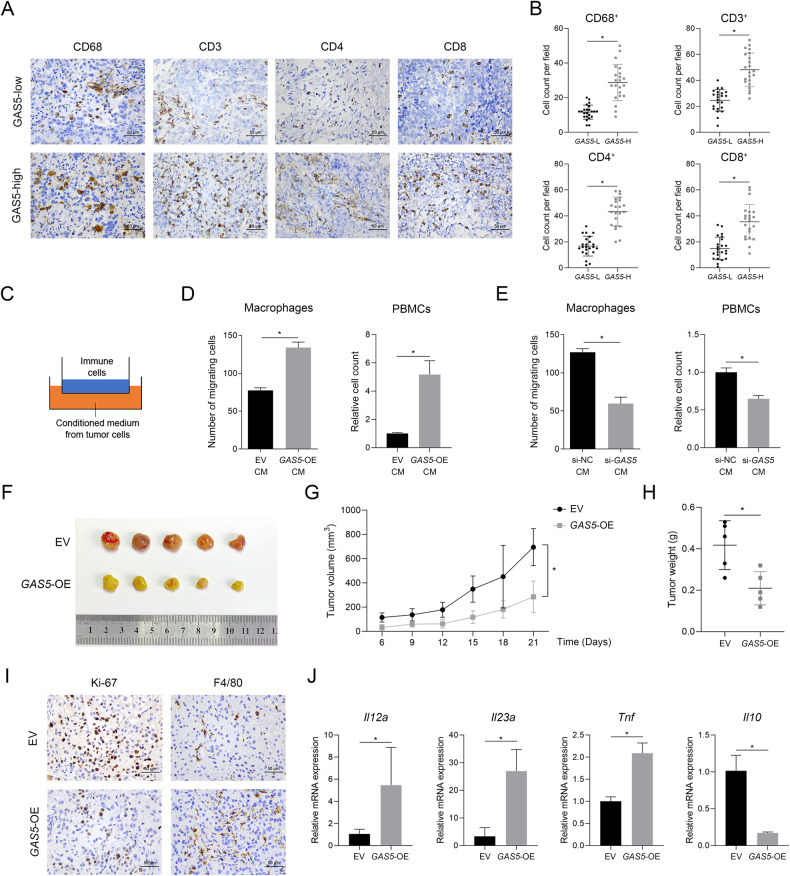
*GAS5* inhibits tumor growth and facilitates immune cell infiltration in NSCLC. Representative images (**A**) and statistical results (**B**) of CD68, CD3, CD4, and CD8 staining in *GAS5*-high and *GAS5*-low NSCLC tissue samples. Scale bar = 50 μm. *GAS5*-low *n* = 23, *GAS5*-high *n* = 22. **C** Graphic illustration of a transwell assay. Immune cells were placed into the upper chamber initially, and the conditioned medium from tumor cells was placed in the lower chamber. **D** More macrophages and PBMCs migrated when the lower chamber was filled with conditioned medium collected from *GAS5*-overexpressing cells. **E** Less macrophages and PBMCs migrated when the lower chamber was filled with conditioned medium collected from *GAS5*-knockdown cells. **F**–**H** *GAS5*-overexpressing cells and control cells were injected subcutaneously into the right flank of each BALB/c nude mouse. Tumor volume and weight were significantly suppressed in the *GAS5*-overexpressing group. *n* = 5 in each group. **I** Representative images of Ki-67 and F4/80 staining in subcutaneous tumors. Scale bar = 50 μm. **J** The expression of *Il12a*, *Il23a*, *Tnf*, and *Il10* analyzed by qPCR in subcutaneous tumors. *n* = 5 in each group. Data are represented as mean ± SD. ^*^P < 0.05.

We had reported that *GAS5* expression was negatively correlated with worse clinical characteristics in NSCLC specimens previously, including tumor size and TNM stage [16], and we analyzed its association with immune cell infiltration in the current study. Firstly, we explored this association using clinical specimens of NSCLC. The expression of *GAS5* was analyzed by qPCR and patients were categorized into *GAS5*-high and *GAS5*-low groups according to the median value. Results of immunohistochemical (IHC) staining showed that higher staining signals of CD68, CD3, CD4, and CD8 were detected in the *GAS5*-high group than in the *GAS5*-low group (Fig. 1A, B), suggesting NSCLC tissues with higher *GAS5* expression levels were infiltrated with more immune cells. Then we adopted transwell assay to evaluate the influence of tumor cell-derived *GAS5* on immune cell recruitment (Fig. 1C). Macrophages and T cells are important parts of TME and can influence immunotherapeutic responsiveness [4, 17, 18]; therefore, THP-1-derived macrophages and PBMCs were chosen. A significant increase in the number of migrated macrophages and PBMCs attracted by conditioned medium from *GAS5*-overexpressing cells was observed (Fig. 1D; Supplementary Fig. 1A). Consistent with this result, fewer macrophages and PBMCs migrated when the lower chamber was filled with conditioned medium from *GAS5*-knockdown cells (Fig. 1E; Supplementary Fig. 1B). To validate the above results in vivo, a xenograft mouse model was established with A549 cells transfected with lentivirus encoding *GAS5* or control lentivirus. Tumor growth was significantly inhibited in mice implanted with *GAS5*-overexpressing cells compared to that in mice implanted with control cells (Fig. 1F–H). The IHC analyses indicated that overexpression of *GAS5* inhibited tumor cell proliferation (Ki-67 staining) and facilitated macrophage infiltration (F4/80 staining) (Fig. 1I; Supplementary Fig. 1C). In addition, we found that the expression of *Il12a*, *Il23a* and *Tnf* was upregulated, while *Il10* was downregulated in *GAS5*-overexpressing subcutaneous tumors (Fig. 1J), indicating a proinflammatory TME. Taken together, our results indicated that tumor cell-derived *GAS5* could enhance immune cell recruitment and suppress tumor development in NSCLC.

### *GAS5* facilitates immune cell infiltration potentially by activating type I interferon signaling pathway

**Fig. 2:**
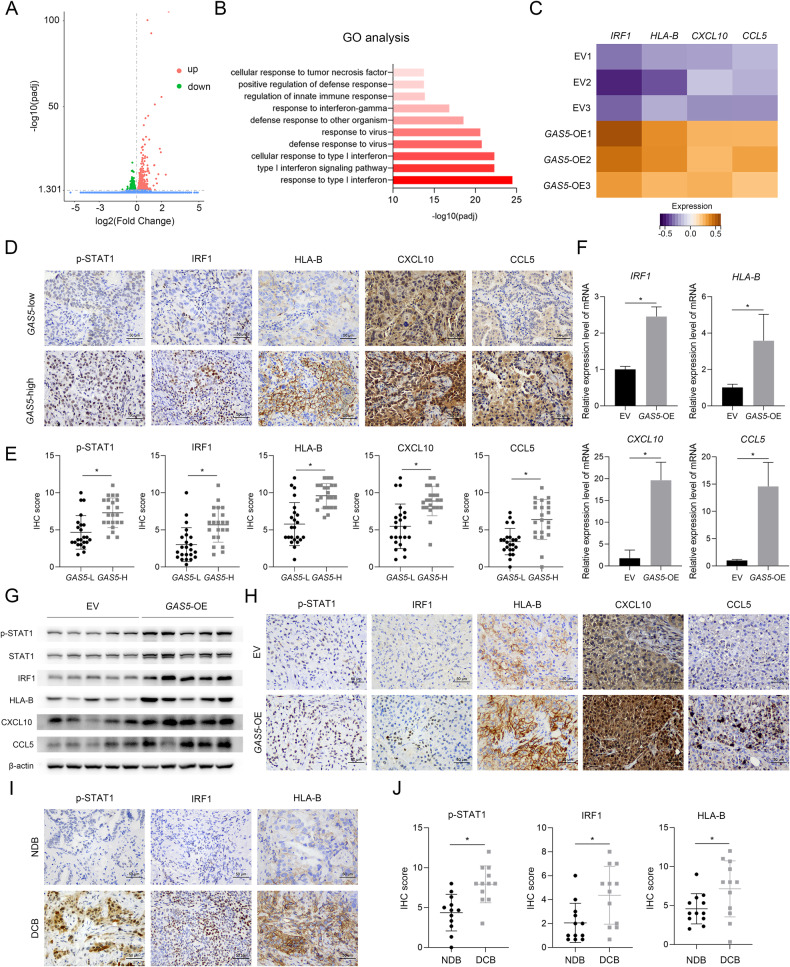
*GAS5* modulates type I interferon signaling in NSCLC. The volcano plot (**A**) and the GO analysis (**B**) of the RNA sequencing of *GAS5*-overexpressing cells and control cells (*n* = 3). **C** The expression profile of *IRF1*, *HLA-B*, *CXCL10*, and *CCL5* in RNA sequencing of *GAS5*-overexpressing cells and control cells. Representative images (**D**) and statistical analyses (**E**) of p-STAT1, IRF1, HLA-B, CXCL10, and CCL5 staining in human NSCLC tissue samples. Scale bar = 50 μm. *GAS5*-low *n* = 23, *GAS5*-high *n* = 22. **F** The mRNA expression of *IRF1*, *HLA-B*, *CXCL10*, and *CCL5* was analyzed by qPCR in subcutaneous tumors. *n* = 5 in each group. **G** The protein level of IRF1, HLA-B, CXCL10, and CCL5 was analyzed by western blot in subcutaneous tumors. *n* = 5 in each group. **H** Representative images of p-STAT1, IRF1, HLA-B, CXCL10, and CCL5 staining in subcutaneous tumors. Scale bar = 50 μm. Representative images (**I**) and statistical results (**J**) of *GAS5*-downstream molecules staining in FFPE specimens of NSCLC patients receiving anti-PD-1/anti-PD-L1 treatment. Scale bar = 50 μm. NDB *n* = 11, DCB *n* = 11. DCB: Durable clinical benefit; NDB no durable benefit. Data are represented as mean ± SD. ^*^P < 0.05.

To explore the underlying mechanism, RNA sequencing was performed in *GAS5*-overexpressing A549 cells and control cells, with 659 genes upregulated and 541 genes downregulated (Fig. 2A). Gene Ontology (GO) enrichment analysis showed that the most upregulated pathways are type I interferon signaling-related (Fig. 2B). The most differentially expressed genes in type I interferon signaling included *IRF1*, *HLA-B*, *CXCL10*, and *CCL5* (Fig. 2C). To explore the association between *GAS5* and the type I interferon signaling pathway, IHC staining was conducted on formalin-fixed, paraffin-embedded (FFPE) specimens of NSCLC. Since the upregulation of signal transducer and activator of transcription 1 (STAT1) phosphorylation is one of the hallmark events during activation of the canonical type I interferon signaling pathway [7], we also examined the level of phosphorylated STAT1 (p-STAT1). The results suggested a higher activation level of the type I interferon signaling in patients in the *GAS5*-high group than in patients in the *GAS5*-low group (Fig. 2D, E). The analyzation of specimens from the xenograft mouse model also supported that *GAS5* could activate type I interferon signaling pathway at both mRNA level (Fig. 2F) and protein level (Fig. 2G, H; Supplementary Fig. 1D). In addition to IRF1, HLA-B, CXCL10, and CCL5, several other molecules in type I interferon signaling, including IFIT2, RSAD2 and IFITM3, were also detected upregulated in *GAS5*-overexpressing subcutaneous tumors (Supplementary Fig. 1E–G).

Immunotherapy has achieved great clinical success and studies have reported tumor immune infiltrates are associated with patients’ response to immunotherapy [18]. We also found that in lung cancer patients receiving neoadjuvant immunotherapy, more infiltrated CD3^+^, CD4^+^, CD8^+^, or CD68^+^ cells were detected in pre-treatment tumor tissues from patients achieving pathologic complete response (pCR) (Supplementary Fig. 1H). Since *GAS5* was identified to influence the infiltration of immune cells and activate type I interferon signaling pathway, we tried to demonstrate the association of the type I interferon signaling and the response of NSCLC patients to immunotherapy. We investigated the protein levels of p-STAT1, IRF1, and HLA-B in these patients by performing IHC staining on the most recent FFPE samples collected before they received anti-PD-1/anti-PD-L1 treatment. Patients who achieved durable clinical benefit (DCB) tended to express higher levels of p-STAT1, IRF1, and HLA-B than patients who received no durable benefit (NDB) (Fig. 2I, J). Taken together, these data suggested that the type I interferon signaling pathway could be activated by *GAS5* in vivo and was correlated with immunotherapy efficacy in NSCLC.

### *GAS5* facilitates immune cell infiltration by activating type I interferon signaling pathway

**Fig. 3:**
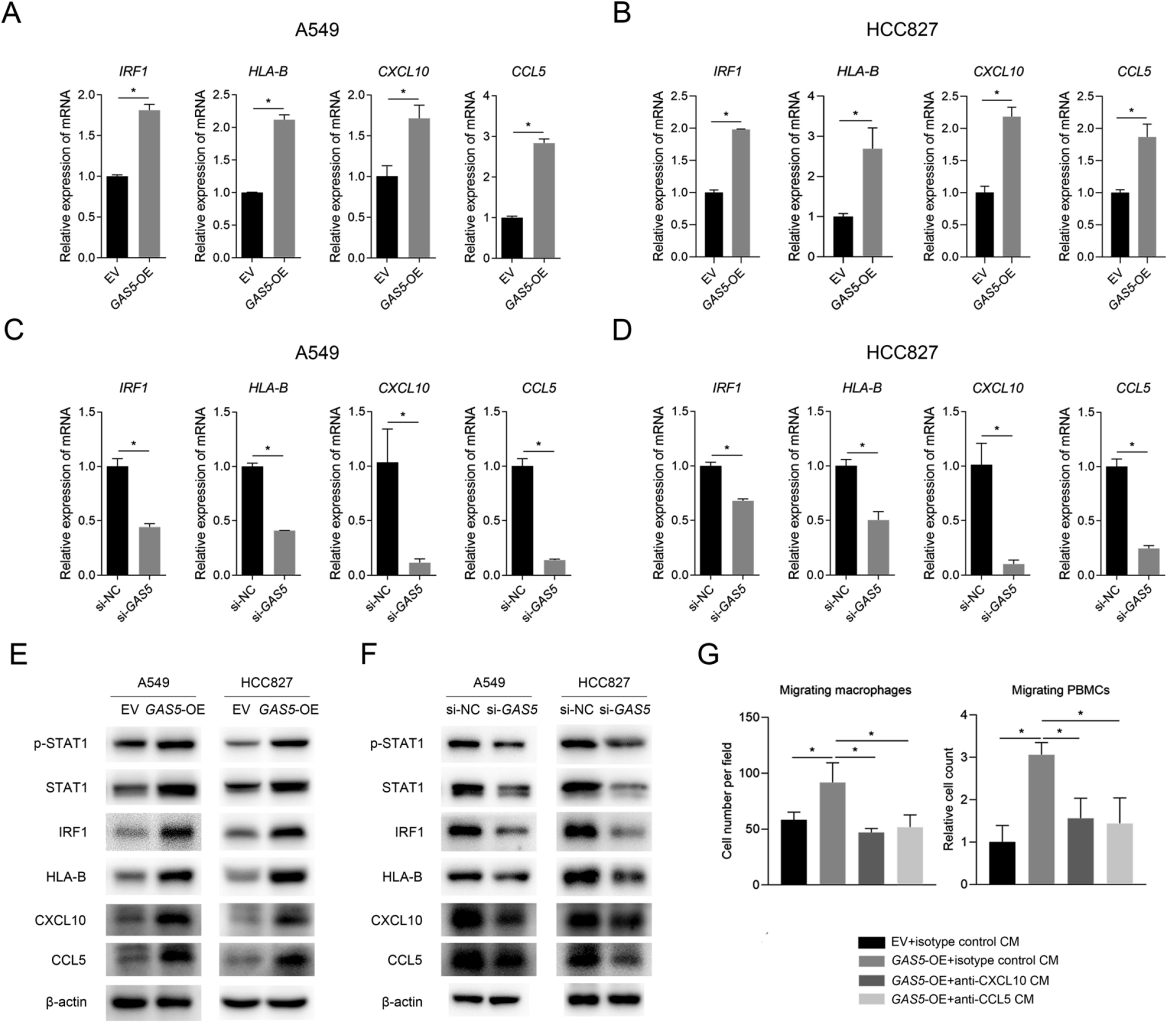
*GAS5* regulates immune cell infiltration via type I interferon signaling. **A**, **B** The mRNA expression of *IRF1*, *HLA-B*, *CXCL10*, and *CCL5* in *GAS5*-overexpressing cells and control cells. **C**, **D** The mRNA expression of *IRF1*, *HLA-B*, *CXCL10*, and *CCL5* in *GAS5*-knockdown and control cells. **E** The protein level of molecules in type I signaling in *GAS5*-overexpressing cells and control cells. **F** The protein level of molecules in type I signaling in *GAS5*-knockdown and control cells. **G** Decreased number of migrated macrophages and PBMCs with the presence of anti-CXCL10 antibody (2.0 μg/ml) or anti-CCL5 antibody (2.0 μg/ml) was observed. Data are represented as mean ± SD. ^*^P < 0.05.

The regulation of type I interferon signaling pathway by *GAS5* was validated in vitro using A549 and HCC827 cells. Results of qPCR showed increased expression of *IRF1*, *HLA-B*, *CXCL10*, and *CCL5* in *GAS5*-overexpressing cells (Fig. 3A, B) and decreased expression of these genes in *GAS5*-knockdown cells (Fig. 3C, D) compared to control cells. The western blot analysis results revealed that the protein levels of p-STAT1, IRF1, HLA-B, CXCL10, and CCL5 were significantly increased by overexpression of *GAS5* (Fig. 3E) and decreased by knockdown of *GAS5* (Fig. 3F). To investigate the role of type I interferon signaling in the recruitment of immune cells by *GAS5*, we added neutralizing antibodies targeting CXCL10 or CCL5, or isotype control into the medium of lower chamber in the transwell assay. Results suggested that the influence of *GAS5* on the migration of macrophages and PBMCs (Fig. 3G, Supplementary Fig. 1I) was partly reversed with the addition of these neutralizing antibodies, indicating that type I interferon signaling mediated tumor cell-derived *GAS5*’s regulatory effect on immune cell infiltration.

### *GAS5* regulates type I interferon signaling by modulating IRF1

**Fig. 4:**
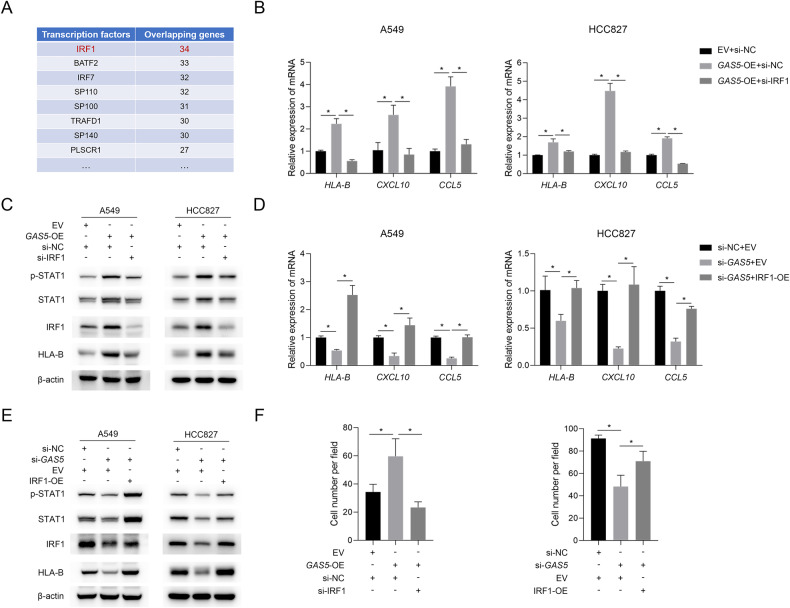
*GAS5* regulates type I interferon signaling by modulating IRF1. **A** The result of the transcription factor enrichment analysis of changed genes in type I interferon signaling using ChEA3. Knockdown of IRF1 downregulated the *GAS5*-overexpression-induced increased mRNA (**B**) and protein (**C**) level of molecules in type I interferon signaling. Overexpression of IRF1 upregulated the *GAS5*-knockdown-induced decreased mRNA (**D**) and protein (**E**) level of molecules in type I interferon signaling. **F** Knockdown or overexpression of IRF1 in tumor cells rescued the *GAS5*-induced changed migration of macrophages. Data are represented as mean ± SD. ^*^P < 0.05.

Since the type I interferon signaling was regulated at the mRNA level and the gene expression can be modulated by transcription factors [19], we tried to identify the transcription factor responsible for the activation of this pathway. The results of transcription factor enrichment analysis performed on ChEA3 [20] showed that IRF1, which is one of the ISGs [21] and can mediate the activation of type I interferon signaling [22, 23] was among the top-ranked potential transcription factors (Fig. 4A). In addition, the level of IRF1 was proven to be modulated by *GAS5* (Figs. 2, 3). Thus, we used a siRNA to reduce the level of IRF1 while overexpressing *GAS5*, and it turned out that the type I interferon signaling activation triggered by overexpression of *GAS5* was attenuated at both the mRNA (Fig. 4B) and protein levels (Fig. 4C). We also increased the level of IRF1 while reducing *GAS5* expression with a siRNA, and the results indicated that the elevation of IRF1 level restored the inhibition of the type I interferon signaling caused by downregulation of *GAS5* (Fig. 4D, E). Moreover, we found that the knockdown of IRF1 counterbalanced the enhancement of macrophage migration by *GAS5*-overexpression, and consistently, overexpression of IRF1 restored the *GAS5*-knockdown mediated decreased migration of macrophages (Fig. 4F). These data suggested that *GAS5* regulates the type I interferon signaling pathway possibly by modulating the level of IRF1.

### MYBBP1A/p53 mediates the regulatory effect of *GAS5* on IRF1

**Fig. 5:**
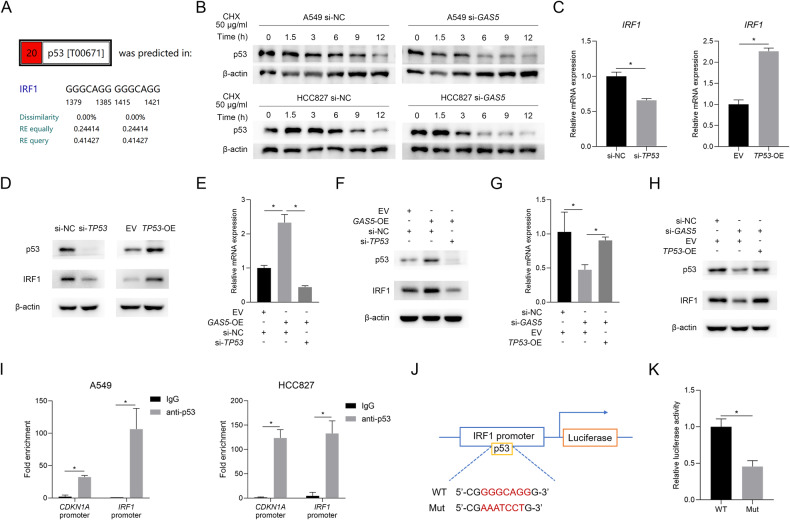
p53 is involved in the regulation of IRF1 by *GAS5.* **A** p53 was predicted as a potential transcription factor of *IRF1* by PROMO. **B** *GAS5*-knockdown cells or control cells were treated with cycloheximide (CHX) for the indicated times and western blot analyses of p53 and β-actin were performed. The expression of *IRF1* was regulated by p53 at the mRNA level (**C**) and protein level (**D**). Knockdown of p53 rescued the *GAS5*-overexpression-induced increased mRNA (**E**) and protein (**F**) level of IRF1. Overexpression of p53 rescued the *GAS5*-knockdown-induced decreased mRNA (**G**) and protein (**H**) level of IRF1. **I** ChIP-qPCR was performed to determine the binding of p53 to the *IRF1* promoter region and *CDKN1A* promoter region. **J** Graphic illustration of the potential p53-binding sequence in the *IRF1* promoter region. **K** The wild-type and mutated luciferase reporter plasmids, p53 plasmid, and Renilla luciferase control plasmid were transfected into HEK293 cells, followed by dual-luciferase reporter assays. Data are represented as mean ± SD. ^*^P < 0.05.

To further demonstrate the mechanism by which *GAS5* regulates the expression of *IRF1*, we predicted potential transcription factors of *IRF1* with PROMO [24] after obtaining the promoter sequence of *IRF1* from the UCSC Genome Browser. The results showed that p53 was one of the possible transcription factors of *IRF1* (Fig. 5A), and our previous study revealed that p53-mediated *GAS5*’s function [16]. Therefore, we chose p53 as the candidate transcription factor. Our previous results had shown *GAS5* regulated p53 at post-transcription level [16], and we confirmed the positive regulatory effect of *GAS5* on p53 protein in both *GAS5*-overexpressing cells and *GAS5*-knockdown cells (Supplementary Fig. 2A). Then, we inhibited protein synthesis using cycloheximide (CHX) and found that degradation of the p53 protein was faster in *GAS5*-knockdown cells (Fig. 5B), indicating that the positive regulatory effect of *GAS5* on p53 was at least partly achieved by an increase in the stability of the p53 protein. After validating the relationship between *GAS5* and p53, we explored whether the effect of *GAS5* on IRF1 is p53-dependent. Firstly, we found p53 could positively regulate the expression of IRF1 at both mRNA level (Fig. 5C) and protein level (Fig. 5D). Second, we confirmed that the decrease of p53 could alleviate *GAS5*’s upregulation effect on IRF1 (Fig. 5E, F), and consistently, the overexpression of p53 could rescue the decreased level of IRF1 by the GAS5-specific siRNA (Fig. 5G, H). Next, the interaction of p53 and *IRF1* promoter region was validated by the analysis of chromatin immunoprecipitation (ChIP) products, and the *CDKN1A* promoter fragment was used as a positive reference (Fig. 5I, Supplementary Fig. 2B). Moreover, the dual-luciferase reporter assays demonstrated that the mutation of binding sites (Fig. 5J) decreased the transcriptional activity of *IRF1* promoter (Fig. 5K). These results indicated that *GAS5* depended on p53 to influence IRF1.

**Fig. 6:**
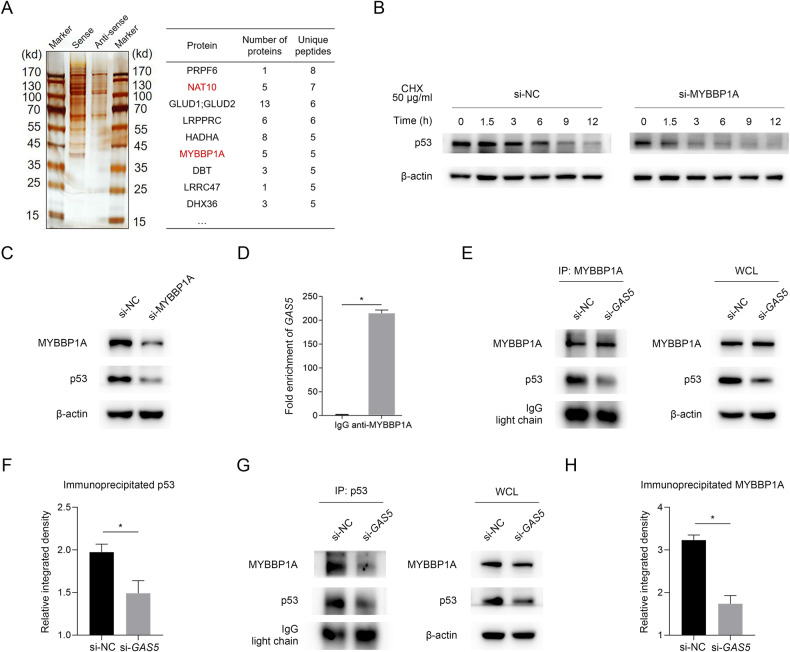
MYBBP1A/p53-mediated the regulatory effect of *GAS5* on IRF1. **A** An RNA pull-down was performed to identify *GAS5*-binding proteins. **B** MYBBBP1A-knockdown cells or control cells were treated with cycloheximide (CHX) for the indicated times and western blot analyses of p53 and β-actin were performed. **C** Level of p53 protein with the knockdown of MYBBP1A. **D** RIP analyses were performed to determine the binding of MYBBP1A protein to *GAS5*. **E** *GAS5*-knockdown or control A549 cells were subjected to co-IP with anti-MYBBP1A antibody and immunoprecipitated proteins were analyzed by western blotting. **F** After quantification of western blotting bands by ImageJ, relative integrated density was calculated using the following formula: relative integrated density = integrated density of p53 in co-IP sample/integrated density of p53 in corresponding group of WCL. **G** *GAS5*-knockdown or control A549 cells were subjected to co-IP with anti-p53 antibody and immunoprecipitated proteins were analyzed by western blotting. **H** After quantification of western blotting bands by ImageJ, relative integrated density was calculated using the following formula: relative integrated density = integrated density of MYBBP1A in co-IP sample/integrated density of MYBBP1A in corresponding group of WCL. WCL whole cell lysate. Data are represented as mean ± SD. ^*^P < 0.05.

Since lncRNAs can mediate signal transduction by interacting with proteins [25], we performed RNA pull-down followed by mass spectrometry to identify *GAS5*-binding proteins that might be involved in the effect of *GAS5* on p53 level, and MYBBP1A was one of the proteins specifically bound to sense *GAS5* but not antisense *GAS5* (Fig. 6A). MYBBP1A is a nucleolar protein that has been reported to bind to transcription factors and regulate their activity [26, 27]. It has been reported that MYBBP1A is able to stabilize the p53 protein and promote p53 activity by directly binding to p53, facilitating the p53-p300 interaction and thus enhancing p53 acetylation [28, 29]. Consistent with previous studies, our results supported that MYBBP1A could stabilize p53 protein by using CHX (Fig. 6B) and reduced level of p53 with knockdown of MYBPP1A was observed (Fig. 6C). To confirm the interaction between *GAS5* and MYBBP1A, an RNA immunoprecipitation (RIP) assay was performed using anti-MYBBP1A antibodies to immunoprecipitate endogenous MYBBP1A together with its bound RNAs from A549 cell lysates. The qPCR results revealed that *GAS5* directly bound to MYBBP1A in A549 cells (Fig. 6D). To evaluate the influence of *GAS5* on the interaction between MYBBP1A and p53, co-immunoprecipitation (co-IP) was conducted using A549 and HCC827 cell lysates with anti-MYBBP1A antibodies or anti-p53 antibodies. Less p53 was detected in *GAS5*-knockown cells than in control cells in the MYBBP1A IP experiment (Fig. 6E, F; Supplementary Fig. 2C, D) and consistently, less MYBBP1A was detected in *GAS5*-knockdown cells than in control cells in the p53 IP experiment (Fig. 6G, H; Supplementary Fig. 2E, F). Taken together, our results suggested that *GAS5* affects the stability of the IRF1 transcription factor p53 by binding to MYBBP1A and therefore regulates the level of IRF1.

### NAT10 regulates *GAS5* expression and mediates its ac4C modification

**Fig. 7:**
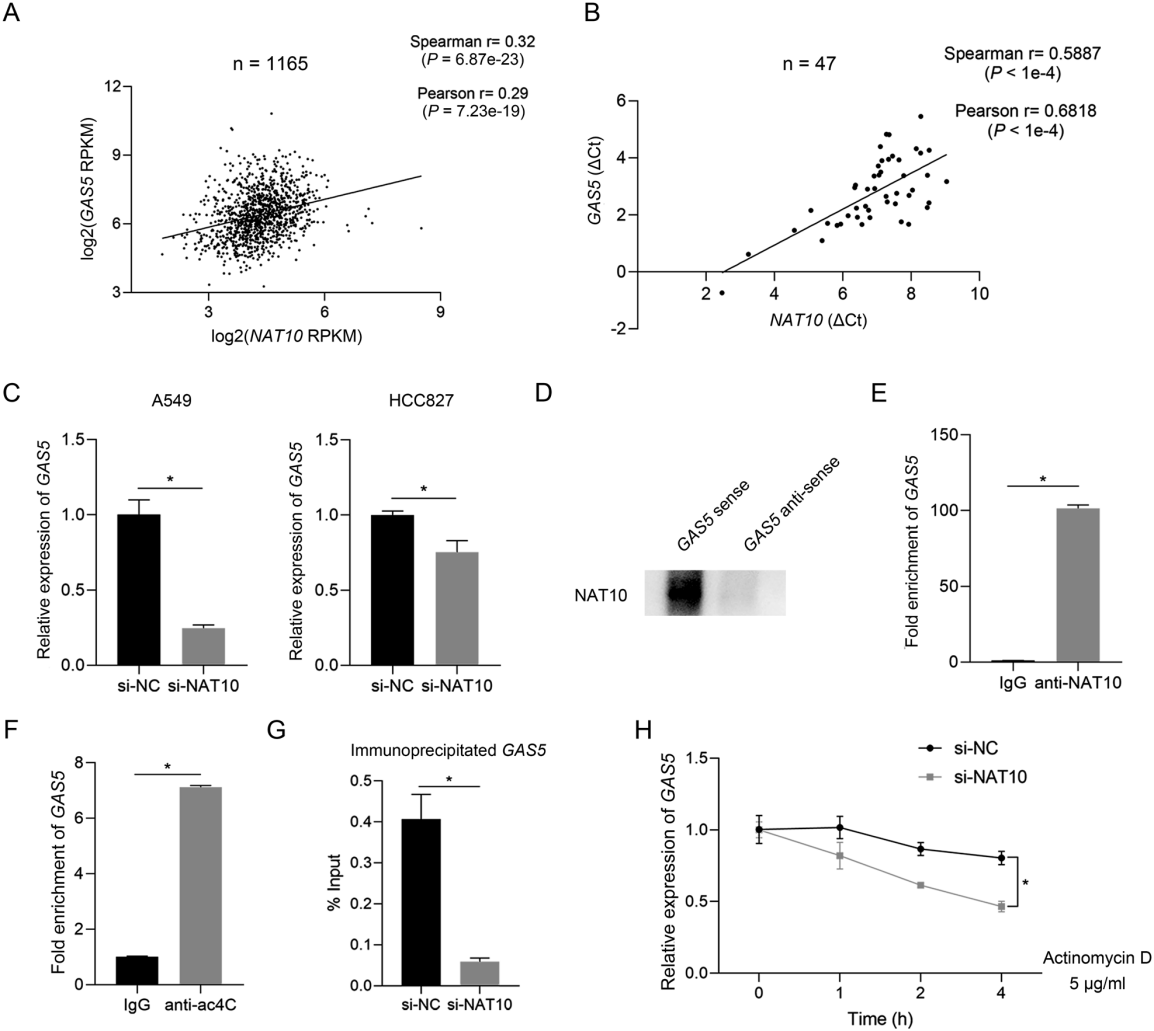
NAT10 regulates *GAS5* expression by mediating ac4C modification and influencing *GAS5* stability. **A** The correlation analysis of *NAT10* and *GAS5* at RNA level in CCLE datasets via cbioportal (*n* = 1165). **B** The correlation analysis of *NAT10* and *GAS5* at RNA level in NSCLC tissue samples (*n* = 47). **C** *GAS5* was downregulated with the knockdown of NAT10. **D** Western blot analysis of RNA pull-down products using anti-NAT10 antibody. **E** RIP analyses were performed to confirm the binding of NAT10 to *GAS5* using anti-NAT10 antibody. **F** RIP analyses were performed to confirm the presence of ac4C modification on *GAS5* using anti-ac4C antibody. **G** The expression of ac4C-modified *GAS5* was explored in NAT10-knockdown cells and control cells using RIP analyses. **H** NAT10-knockdown cells or control cells were treated with Actinomycin D for the indicated time and qPCR analyses of *GAS5* and β-actin were performed. Data are represented as mean ± SD. ^*^P < 0.05.

The expression of lncRNAs can be regulated by their binding proteins [30]. The results of mass spectrometry performed after RNA pull-down showed that NAT10 bound to *GAS5* (Fig. 6A). NAT10 had been reported to influence the RNA expression by mediating ac4C modification [31, 32]; thus, we tried to determine the association of NAT10 and *GAS5*. Clinically, the analysis of CCLE dataset showed that the RNA expression of *NAT10* and *GAS5* was significantly positively correlated in tumor cells (Fig. 7A). A positive association of *NAT10* and *GAS5* was also found in clinical specimens of NSCLC (Fig. 7B). Then, we explored whether NAT10 could regulate the expression of *GAS5*, and the results showed that the expression of *GAS5* was decreased with the knockdown of NAT10 in NSCLC cells (Fig. 7C). After confirming the correlation of *GAS5* and NAT10, we validated the interaction of NAT10 and *GAS5* by western blot analysis (Fig. 7D). Consistently, the qPCR results of the RIP assay products precipitated with the anti-NAT10 antibody showed significant enrichment of *GAS5* in the anti-NAT10 antibody group compared to the IgG group (Fig. 7E). Previous studies suggested that ac4C modification plays an important role in NAT10’s function [33], and we also identified that ac4C modification was present by performing RIP using anti-ac4C antibodies (Fig. 7F). Moreover, less ac4C-modified *GAS5* was detected in NAT10-knockdown cells, suggesting NAT10-mediated ac4C modification of *GAS5* (Fig. 7G). We also treated cells with actinomycin D to inhibit RNA transcription and found that the reduction in NAT10 level significantly accelerated the decay of *GAS5* in A549 (Fig. 7H), indicating that NAT10 regulates *GAS5* stability. Taken together, these data suggested that NAT10 positively regulates *GAS5* level via ac4C modification.

## Discussion

The TME is essential for tumor development and progression [34], and the ‘hot tumors’ phenotype of the tumor immune microenvironment predicts a better response to immunotherapy [18]. Previous studies have shown the role of tumor cell-derived lncRNAs in the regulation of immune cell infiltration [35, 36]. Our study demonstrated that *GAS5* was positively correlated with immune cell infiltration in NSCLC and it enhanced the recruitment of macrophages and T cells by activating type I interferon signaling pathway via MYBBP1A-p53/IRF1 axis in tumor cells. We also found that tumor tissues of patients who achieved durable clinical benefit from immunotherapy tended to exhibit higher levels of type I interferon signaling. In this context, the analysis of *GAS5* would help distinguish patients who are more likely to benefit from anti-PD-1/anti-PD-L1 treatment in NSCLC, and the strategy of upregulating *GAS5* or type I interferon signaling might improve immunotherapy efficacy for low *GAS5*-expressing patients by altering tumor immune microenvironment.

Type I interferon signaling plays a fundamental role in anti-tumor immunity and closely correlates with disease outcomes in patients with cancer [8, 37]. Type I interferon signaling is activated in tumors with inflamed phenotypes, especially in malignant cells [38] and its effect on immune cell infiltration is mediated by augmenting the secretion of cytokines [39, 40]. DNA mismatch repair-deficient colorectal cancer cells can overexpress CXCL10 and CCL5 via endogenous activation of type I interferon signaling, thus increasing the recruitment of CD8^+^ T cells into tumors [39]. In addition, type I interferon signaling also controls the infiltration of γδT17 cells in breast cancer via IL7 [40]. We found that *GAS5* positively modulated the level of CXCL10 and CCL5 by regulating the type I signaling pathway and facilitated the recruitment of macrophages and T cells in NSCLC. Previous studies have indicated that the activation of type I interferon signaling can boost the response to anti-PD-1/anti-PD-L1 therapy in various cancers by upregulating antigen presentation, converting ‘cold tumors’ to ‘hot tumors’ and increasing PD-L1 expression [6, 41], including NSCLC [42–44]. Due to systemic side effects caused by type I interferons, strategies that induce type I interferon signaling have been explored as immunotherapy combination partners, including small molecule inhibitors or agonists [45, 46], cryoablation [44] and radiation therapy [47], and these studies revealed promising synergistic effect of these strategies with anti-PD-1/anti-PD-L1 therapy in cancer treatment. In our study, the expression of *GAS5*-downstream molecules in type I interferon signaling was higher in patients who were able to achieve DCB than in patients who could not achieve DCB, indicating that the downregulation of *GAS5* in NSCLC might be correlated with worse prognosis in NSCLC patients receiving anti-PD-1/anti-PD-L1 treatment. While the results of H. Wang’s [42] and our study supported that NSCLC patients with higher expression of type I interferon-related genes benefited more from immunotherapy, P. Trono’s research suggested different results [10]. Since this discrepancy may be caused by a small sample size, different detection methods, and different evaluation criteria, studies with large sample sizes and consistent study designs are required in the future.

IRF1 has been identified as a transcriptional activator of the type I interferon system and a suppressor of oncogenesis [48, 49]. TNF receptors in microvascular endothelial cells can signal through IRF1 to activate the type I interferon pathway and recruit monocytes [22]. We found that *GAS5* modulates IRF1 at the transcriptional level by influencing p53, a transcription factor of IRF1, which in turn affects the activation of the type I interferon pathway. The restoration of p53 activity was found to enhance type I interferon signaling and promote T cell infiltration by inducing immunogenic cell death [50]. In our study, we found that p53 can bind to the promoter region of *IRF1*, positively regulate the expression of *IRF1*, and then activate type I interferon signaling. MYBBP1A was first identified for its binding to the negative regulatory domain of MYB [27]. Studies have shown that MYBBP1A can bind to p53 and enhance p53 acetylation, therefore influencing the stability and activity of p53 [28, 29, 51]. LncRNAs may play a role in the regulation of the function of MYBBP1A via direct interaction. The lncRNA *DRHC* can inhibit MEK/ERK signaling by binding to MYBBP1A and influencing the MYBBP1A-MYB interaction [52]. The lncRNA *PURPL* interacts with MYBBP1A and can suppress the expression of p53 by interfering with the MYBBP1A-p53 interaction [53]. Similarly, *GAS5* can enhance the association of MYBBP1A and p53 by directly binding to MYBBP1A, thus influencing the stability of p53.

RNA can be epigenetically modified by more than 170 types of modifications and these modifications may regulate RNA fate [54]. The ac4C modification was initially identified on tRNA and rRNA and has been reported in recent years to exist on mRNA and regulate RNA decay and translation [55]. Recently, ac4C modification was detected on a lncRNA, which increased the stability and expression of the lncRNA [56], suggesting a promising role of ac4C modification in the regulation of lncRNA. So far, NAT10 is the only acetyltransferase known to catalyze ac4C modification, and current research on ac4C modification and NAT10 mainly focuses on mRNA in multiple physiological and pathological conditions, including self-renewal of embryonic stem cells [57], metastasis [32, 58] and drug resistance [59] of the tumor, osteogenesis of BMSCs [60] and human immunodeficiency virus 1 infection [61]. Compared with the research on mRNA, the number of studies on ac4C modification and lncRNA is rather less. In this study, by conducting RNA pull-down assay, mass spectrometry, ac4C-RIP, and RNA stability assay, we found that NAT10 bound to lncRNA *GAS5*, mediated its ac4C modification, and regulated its stability. Moreover, analyses of clinical specimens confirmed the relationship between NAT10 and *GAS5*.

There are several limitations in our study. First, the BALB/c nude mice used in this study are immunodeficient mice lacking T cells, while T cells are an important component of the TME and are closely related to the efficacy of immunotherapy. In this case, a model with humanized mice is needed to improve the exploration of the effects of *GAS5* on the TME and the efficacy of immunotherapy. Second, more efficient strategies of upregulating GAS5, such as small molecule agonists, are needed to evaluate its efficacy as a combined therapeutic target for immunotherapy in vivo. Third, research on the detailed mechanisms of how NAT10 regulates *GAS5* and how *GAS5* influences the interaction of MYBBP1A and p53 may help understand GAS5-mediated responses more comprehensively. For example, a more advanced technique [62] may be needed to identify the specific site of ac4C modification on *GAS5*. Additionally, more cases are required to validate the efficiency of *GAS5* as a biomarker for discriminating immunotherapy responders in NSCLC.

In summary, our study identified a pivotal role of *GAS5*, which was stabilized by NAT10-mediated ac4C modification, in modulating TME via MYBBP1A-p53/IRF1/type I interferon signaling axis in NSCLC. *GAS5* expression may indicate the phenotype of immune cell infiltration and help to screen responders to anti-PD-1/PD-L1 treatment. Strategies of upregulating *GAS5* are promising therapeutic candidates for reshaping ‘cold tumors’ into ‘hot tumors’. Therefore, our findings suggest that *GAS5* can be an ideal predictive biomarker for anti-PD-1/PD-L1 treatment and a novel therapeutic target for improving the efficacy of immunotherapeutic regimens in NSCLC.

## Materials and methods

### Cell lines and transfection

The human non-small cell lung cancer cell lines A549 and HCC827, human embryonic kidney 293 (HEK293) cells, and the human monocyte cell line THP-1 were purchased from the Institute of Biochemistry and Cell Biology, Chinese Academy of Sciences (Shanghai, China). The purchased cell lines were validated by short tandem repeat analysis and tested for mycoplasma contamination within the past 6 months. Peripheral blood mononuclear cells (PBMCs) were isolated from the volunteer’s blood using a lymphocyte separation solution (FCMACS, Nanjing, China). The A549 cell line, HCC827 cell line, and HEK293 cell line were maintained in F-12K (Meilun Bio, Dalian, China), RPMI-1640 (Gibco, Carlsbad, California), and DMEM (Gibco, Carlsbad, California) medium, respectively, supplemented with 10% FBS (HyClone, Carlsbad, California). The THP-1 cell line was cultured in RPMI-1640 with 10% FBS and 0.05 mM β-mercaptoethanol (Procell, Wuhan, China). All the cell lines were maintained in a humidified environment at 37 °C in 5% CO_2_. For differentiation into macrophages, THP-1 cells were maintained in RPMI-1640 medium supplemented with 1% FBS and 100 ng/ml phorbol-12-myristate acetate (FCMACS, Nanjing, China) for 24 h, and the culture medium was then replaced with RPMI-1640 medium supplemented with 10% FBS for another 24 h.

Plasmid and siRNA transfection was performed using X-tremeGENE HP DNA Transfection Reagent (06366546001, Roche) and jetPRIME (114-15, Polyplus-transfection, Illkirch, France), respectively, following the manufacturers’ protocols. The detailed sequences of the siRNAs are listed in Supplementary Table 1. Since si-NC was purchased from RiboBio (Guangzhou, China) directly, the sequence was not provided. To generate cells with stable *GAS5*-overexpression, the *GAS5*-overexpression and empty vector lentiviruses were purchased from HanBio (Shanghai, China). For lentiviral transduction, A549 cells were cultured in a complete medium containing lentivirus in the presence of 6 μg/mL polybrene, and after 24 h, the medium was replaced with a normal complete culture medium. After selection with 4 μg/mL puromycin (Solarbio, Beijing, China), the stable GAS5-overexpressing A549 cells and control cells were used for the following studies.

### Tissue samples

A total of 47 lung cancer tumor tissues were obtained from Jinling Hospital. Some of these tissues were fixed with formalin and then embedded in paraffin, and the others were rapidly frozen in liquid nitrogen for RNA extraction and other experiments. FFPE lung cancer tissues from 24 patients who had received anti-PD-1/anti-PD-L1 therapy were also obtained from Jinling Hospital. The efficacy of anti-PD-1/anti-PD-L1 therapy was defined as DCB (complete response or partial response or stable disease lasting over 6 months) or NDB (progressive disease or stable disease lasting no longer than 6 months) [63]. In addition, FFPE lung cancer tissues from 17 patients who received neoadjuvant immunotherapy were also retrospectively collected from Jinling Hospital. The achievement of a pathologic complete response was evaluated by pathologists. This study was approved by the Ethics Committee of Jinling Hospital, and tissue samples were collected in compliance with the informed consent policy.

### RNA extraction and qPCR

To extract RNA from tissues, tissues were homogenized in TRIzol reagent (Vazyme, Nanjing, China) with beads and were then placed on ice for 30 min. After that, ice-cold chloroform, isopropanol, and 75% ethanol were used sequentially for RNA extraction. To extract RNA from cultured cells, the same protocol was followed, except that the step of homogenization with beads was omitted. Reverse transcription was performed with HiScript III RT SuperMix for qPCR (Vazyme, Nanjing, China) and the expression levels of targeted genes were evaluated via qPCR with ChamQ SYBR Color qPCR Master Mix (Vazyme, Nanjing, China). β-actin was used as the internal reference gene. The primer sequences are listed in Supplementary Table 1.

### Western blot analysis and co-IP

Cells were homogenized using RIPA buffer with protease inhibitor cocktail (Bimake, Houston, Texas) on ice for 20 min. Then, cell lysates were centrifuged at 12,000 rpm for 15 min at 4 °C, and supernatants were collected for western blot analysis or co-IP with the indicated antibodies (Supplementary Table 2). Western blot signals were detected using ECL Ultra Western HRP Substrate (Millipore, Darmstadt, Germany).

For co-IP, supernatants of cell lysates were incubated with the indicated antibodies or control IgG in RIPA buffer supplemented with protease inhibitor cocktail with rotation at 4 °C overnight, and Protein A/G PLUS-Agarose beads (Santa Cruz, Dallas, Texas) were then added and incubated with rotation for 4 more hours. Then, the beads with bound protein complexes were washed with RIPA buffer 3 times and eluted with 2× SDS loading buffer at 100 °C for 10 min. The eluted protein was analyzed by western blotting. Quantifications of western blotting bands were achieved using ImageJ software (for Windows, USA).

### ChIP

ChIP was performed using a Magna ChIP Kit (17-10086, Millipore, Darmstadt, Germany) according to the manufacturer’s protocol. Briefly, 1 × 10^7^ cells were fixed with 1% formaldehyde, lysed, and sonicated. Then, an anti-p53 antibody or control rabbit IgG was added to the sheared cross-linked chromatin with protein A/G magnetic beads and rotated overnight at 4 °C. After elution and reverse cross-linking and purification, the immunoprecipitated chromatin DNA was quantified by qPCR or DNA agarose electrophoresis. *CDKN1A* was used as a positive control. The sequences of primers targeting p53-binding sites for *IRF1* promoter and *CDKN1A* promoter are listed in Supplementary Table 1.

### RIP

RIP was performed following the instructions of the EZ-Magna RIP Kit (17-701, Millipore, Darmstadt, Germany). Briefly, cells were harvested and were then lysed in a complete RIP Lysis Buffer, and magnetic beads were prepared. Then, the antibody of interest or negative control IgG was incubated with the magnetic beads for 30 min with rotation at room temperature, and cell lysate supernatants were then added and incubated with rotation overnight at 4 °C. The magnetic beads with bound protein-RNA complexes were washed 6 times, and the captured RNA was then freed from beads by proteinase K digestion and purified by phenol: chloroform: isoamyl alcohol. After being precipitated overnight at −80 °C, RNA was extracted and analyzed via qPCR.

### RNA pull-down assay

The pcDNA3.1-*GAS5*-sense and pcDNA3.1-*GAS5*-antisense constructs were linearized by digestion with the BamHI restriction enzyme (FD0054, Thermo Scientific, Waltham, Massachusetts), and in vitro transcription was then performed using a mMESSAGE mMACHINE™ T7 Transcription Kit (AM1344, Invitrogen, Waltham, Massachusetts). Transcribed RNAs were purified by a MEGAclear™ Transcription Clean-Up Kit (AM1908, Invitrogen, Waltham, Massachusetts) and were then biotin-labeled with a Pierce™ RNA 3’ End Desthiobiotinylation Kit (20163, Thermo Scientific, Waltham, Massachusetts). RNA pull-down was performed using a Pierce™ Magnetic RNA-Protein Pull-Down Kit (20164, Thermo Scientific, Waltham, Massachusetts). Briefly, labeled RNAs were captured by magnetic beads in a capture buffer with rotation at room temperature for 30 min. Then, prepared cell lysates were rotated with RNA-magnetic bead complexes in RNA-protein binding buffer at 4 °C for 60 min. After washing 3 times, the precipitated products were eluted in an elution buffer and subjected to mass spectrometry analysis or western blot analysis.

### RNA sequencing

For RNA sequencing, stable *GAS5*-overexpressing A549 cells and control cells were collected for RNA extraction. After checking the purity and integrity of RNA, a total amount of 1 µg RNA per sample was used as input material for the generation of sequencing libraries using NEBNext UltraTM RNA Library Prep Kit for Illumina (NEB, USA) following the manufacturer’s recommendations. The libraries were sequenced on an Illumina Novaseq platform and 150 bp paired-end reads were generated at Novogene Co., Ltd. Paired-end clean reads were aligned to the reference genome using Hisat2 v2.0.5. FPKM, the expected number of Fragments Per Kilobase of transcript sequence per Million base pairs sequenced, was used for estimating gene expression. Differential expression analysis was performed using the DESeq2 R package (1.16.1). The *P*-values were adjusted using Benjamini and Hochberg’s approach and genes with an adjusted *P*-value < 0.05 were considered as differentially expressed. GO enrichment analysis of differentially expressed genes was conducted via the clusterProfiler R package.

### Transwell assay

Briefly, 1 × 10^5^ THP-1 cells were seeded into the upper chamber (3422, Corning, New York, New York) and were then induced to differentiate into macrophages by PMA as described in the ‘Cell lines and transfection’ section. Then, the medium in the lower chamber was replaced with conditioned medium collected from tumor cells, and the medium in the upper chamber was replaced with RPMI-1640. After 24 h, the Transwell membrane was fixed with methanol and stained with crystal violet (C0121, Beyotime, Suzhou, China). To count the cells that had migrated, a microscope (Zeiss, Oberkochen, Germany) was used to image the fields of view. For PBMCs, 1 × 10^5^ PBMCs in 1640 containing 1% FBS were placed in the upper chamber (MCMP24H48, Millipore, Darmstadt, Germany), and the lower chamber was filled with conditioned media from tumor cells. After 3-4 h, cells that migrated into the lower chamber were counted using a cell counting chamber. For CXCL10 and CCL5 neutralizing experiments, anti-CXCL10 antibody (2.0 μg/ml) and anti-CCL5 antibody (2.0 μg/ml) were added to the collected conditioned medium 30 min before the migration assay started.

### IHC staining and immunofluorescence staining

FFPE tissue samples were deparaffinized in xylene and rehydrated in ethanol. After heat-induced epitope retrieval, IHC staining was conducted using anti-CD68, anti-F4/80, anti-CD3, anti-CD4, anti-CD8, anti-IRF1, anti-p-STAT1, anti-HLA-B, anti-CXCL10, anti-CCL5, anti-IFIT2, anti-RSAD2, and anti-IFITM3 antibodies. Immunofluorescence staining was performed using anti-CD68, anti-CD163, anti-CD3, anti-CD4 and anti-CD8 antibodies. For IRF1, p-STAT1, and HLA-B, CXCL10, and CCL5 staining, the score of the percentage of positive cells (0, 0–5%; 1, 6–25%; 2, 26–50%; 3, 51–75%; 4, >75%) and the score of the intensity of positive staining (0, negative; 1, week; 2, moderate; 3, strong) were multiplied to evaluate the expression of the molecules of interest. For CD68, CD3, CD4, CD8, and F4/80 staining, the number of positive cells in the fields of view was counted to assess the infiltration level of corresponding immune cells.

### Dual-luciferase reporter assay

The wild-type and mutated promoter sequences of *IRF1* were synthesized and then cloned into Pgl4.10 to construct the luciferase reporter plasmids, respectively. HEK293 cells were seeded into 24-well plates and were transiently transfected with the luciferase reporter plasmids, p53 plasmid, and Renilla luciferase control plasmids. The luciferase activities were detected using the Dual-Luciferase Assay kit (E1910, Promega) following the manufacturer’s protocol. The relative luciferase activity was obtained by normalizing the firefly luciferase activity to the corresponding Renilla luciferase activity.

### In vivo study

Four- to six-week-old female BALB/c nude mice were purchased from GemPharmatech (Nanjing, China). A total of 3 × 10^6^ stable GAS5-overexpressing A549 cells or control A549 cells in 60 μL PBS (mixed with 40 μL Matrigel) were injected subcutaneously into the right flank of each mouse using an insulin syringe (KDL, Wenzhou, China). The tumor size was measured by a caliper every 3 days after 6 days of injection, and the tumor volume was calculated using the following formula: 1/2 (length × width^2^). Mice were humanely sacrificed on Day 21 and tumors were carefully isolated. The harvested tumors were weighed, photographed, and then fixed with formalin for IHC staining or rapidly frozen in liquid nitrogen for RNA and protein assays. To reduce the influence of individual differences on the experimental results, the same species, strain, sex, and age of animals were selected in this study and their weights were similar when measured before the injection of tumor cells. The operators who injected tumor cells into mice were blinded to the group allocation when they operated. All animal experiments were performed in accordance with a protocol approved by the Animal Care and Use Committee of Jinling Hospital.

### Bioinformatics analysis

To identify transcription factors responsible for the activation of type I interferon signaling, ChEA3 (https://maayanlab.cloud/chea3/) was used. Potential transcription factors of IRF1 were obtained via PROMO (https://alggen.lsi.upc.es/cgi-bin/promo_v3/promo/promoinit.cgi?dirDB=TF_8.3). The association of *NAT10* and *GAS5* expression was assessed in CCLE datasets via cbioportal (http://www.cbioportal.org/).

### Statistical analysis

All in vitro experiments were repeated in triplicate. Statistical analyses were performed using GraphPad Prism 8.0 (GraphPad Software, San Diego, California). Data were plotted using GraphPad Prism 8.0. Two-tailed Student’s *t*-test was used to compare the differences between the two groups. Pearson correlation analysis and Spearman correlation analysis were both conducted to analyze correlations between two groups. A *P*-value < 0.05 was considered statistically significant.

### Supplementary information

Supplementary information

Original western blots

## Data Availability

The data generated or analyzed during the current study are available from the corresponding authors upon reasonable request.
