# Supplementary material for: NAT10-mediated upregulation of GAS5 facilitates immune cell infiltration in non-small cell lung cancer via the MYBBP1A-p53/IRF1/type I interferon signaling axis
Source: Cell Death Discov. 2024 May 18;10:240. doi: 10.1038/s41420-024-01997-2 (PMC11102450; doi:10.1038/s41420-024-01997-2)
Supplement: Supplementary file 1 — Supplementary information [file 41420_2024_1997_MOESM1_ESM.docx]

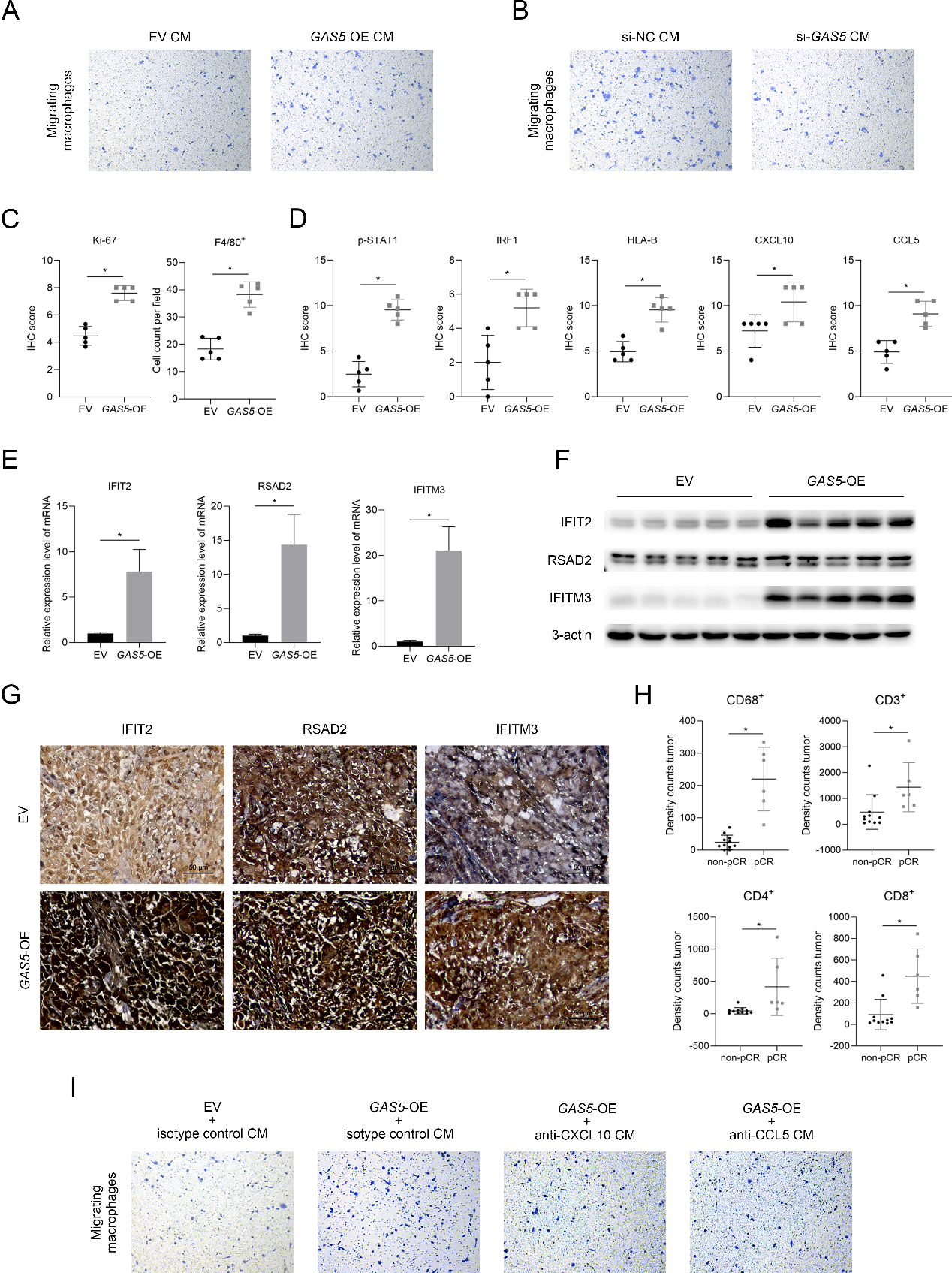


**Supplementary figure 1. A**, **B** Representative images of migrating THP-1-derived macrophages attracted by conditioned medium of tumor cells in the transwell assay. **C** Statistical analyses of Ki-67 staining and F4/80^+^ cells in subcutaneous tumors. n=5 in each group. **D** Statistical analyses of p-STAT1, IRF1, HLA-B, CXCL10 and CCL5 staining in subcutaneous tumors. n=5 in each group. **E** The mRNA expression of *IFIT2*, *RSAD2* and *IFITM3* analyzed by qPCR in subcutaneous tumors. n=5 in each group. **F** The protein level of IFIT2, RSAD2 and IFITM3 analyzed by western blot in subcutaneous tumors. n=5 in each group. **G** Representative images of IFIT2, RSAD2 and IFITM3 staining in subcutaneous tumors. Scale bar = 50 μm. **H** The counts of CD68^+^ cells, CD3^+^ cells, CD4^+^ cells and CD8^+^ cells in pretreatment tumor tissues of NSCLC patients receiving neoadjuvant chemo-immunotherapy. Non-pCR n=11, pCR n=6. **I** Representative images of migrating THP-1-derived macrophages attracted by conditioned medium of tumor cells supplemented with anti-CXCL10/CCL5 antibody (2.0 μg/ml) or isotype control in the transwell assay. pCR, pathologic complete response. Data are represented as mean ± SD. ^*^*P*<0.05.


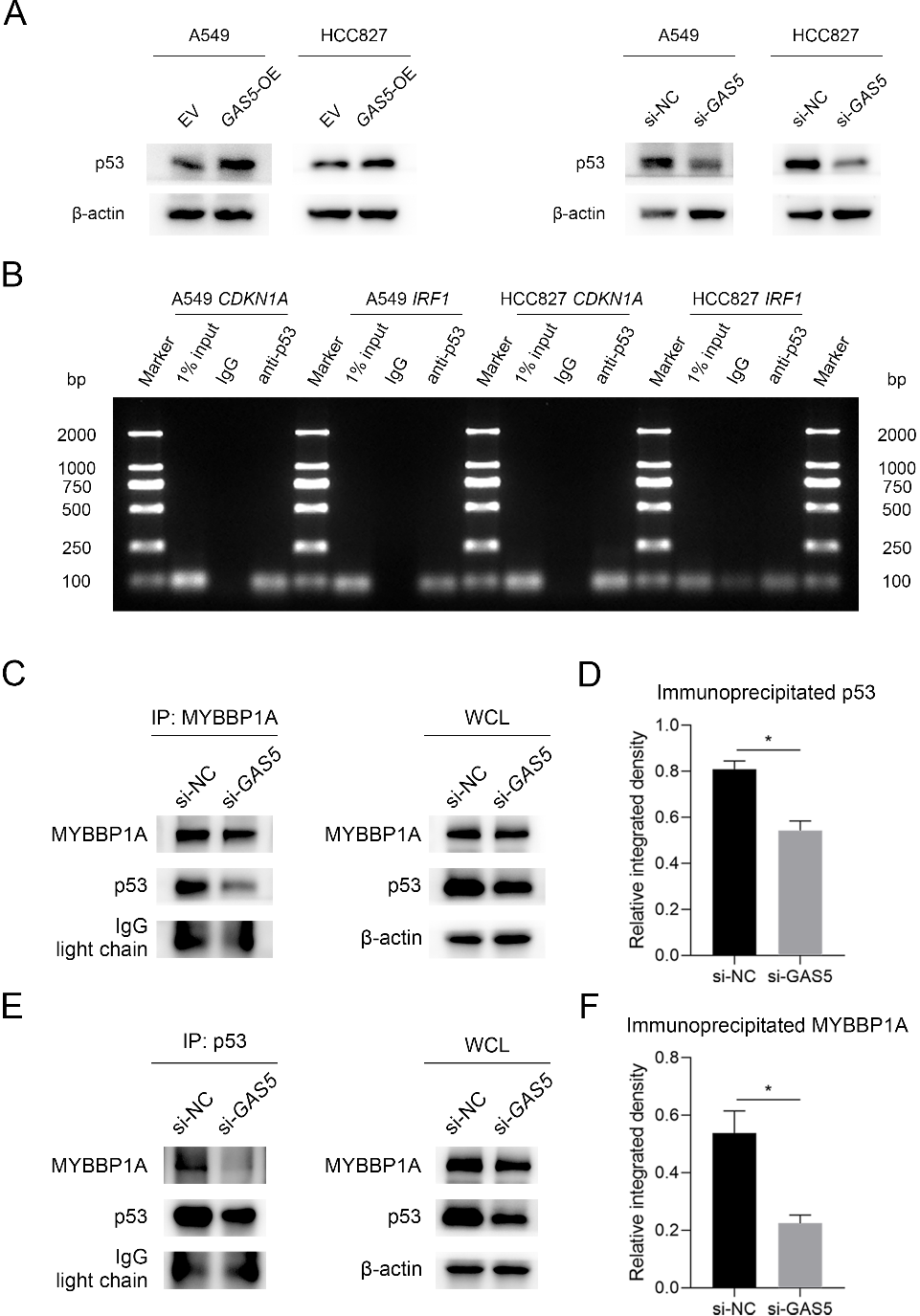


**Supplementary figure 2. A** The protein level of p53 was upregulated by increasing *GAS5* expression and was downregulated by decreasing *GAS5* expression in tumor cells. **B** The image of DNA agarose electrophoresis of DNA fragments immunoprecipitated by anti-p53 antibody. The *CDKN1A* promoter fragment was used as a positive reference. **C** *GAS5*-knockdown or control HCC827 cells were subjected to co-IP with anti-MYBBP1A antibody and immunoprecipitated proteins were analyzed by western blotting. **D** After quantification of western blotting bands by ImageJ, relative integrated density was calculated using the following formula: relative integrated density = integrated density of p53 in co-IP sample/integrated density of p53 in corresponding group of WCL. **E** *GAS5*-knockdown or control HCC827 cells were subjected to co-IP with anti-p53 antibody and immunoprecipitated proteins were analyzed by western blotting. **F** After quantification of western blotting bands by ImageJ, relative integrated density was calculated using the following formula: relative integrated density = integrated density of MYBBP1A in co-IP sample/integrated density of MYBBP1A in corresponding group of WCL. WCL, whole cell lysate. Data are represented as mean ± SD. ^*^*P*<0.05.

**Supplementary Table 1** Oligonucleotide sequences used in this study.

| **Oligonucleotide sequences (5’-3’)** | |
| --- | --- |
| **si-RNAs** | |
| si-*GAS5* | CUUGCCUGGACCAGCUUAAUU |
| si-TP53 | GUAAUCUACUGGGACGGAATT |
| si-NAT10 | AUGGAACACUGAACAUAAATT |
| si-MYBBP1A | GGUCCGAGAUGAAAUAUGCCCUGAA |
| si-IRF1 | GCGTGTCTTCACAGATCTG |
| **Primers for ChIP-qPCR** | |
| *IRF1* F | CAGCGGCCAAGTTGGGATTT |
| *IRF1* R | GGAAGTGAAGAAGGCAGAAAGC |
| *CDKN1A* F | AGCAGGCTGTGGCTCTGATT |
| *CDKN1A* R | CAAAATAGCCACCAGCCTCTTCT |
| **Primers for qPCR** | |
| Human *CXCL10* F | GTGGCATTCAAGGAGTACCTC |
| Human *CXCL10* R | TGATGGCCTTCGATTCTGGATT |
| Human *TP53* F | CAGCACATGACGGAGGTTGT |
| Human *TP53* R | TCATCCAAATACTCCACACGC |
| Human *NAT10* F | ATAGCAGCCACAAACATTCGC |
| Human *NAT10* R | ACACACATGCCGAAGGTATTG |
| Human *ACTB* F | ACGCCAACACAGTGCTGTCTG |
| Human *ACTB* R | GGCCGGACTCGTCATACTCC |
| Human *GAS5* F | CTTGCCTGGACCAGCTTAAT |
| Human *GAS5* R | CAAGCCGACTCTCCATACCT |
| Human *IRF1* F | CTGTGCGAGTGTACCGGATG |
| Human *IRF1* R | ATCCCCACATGACTTCCTCTT |
| Human *HLA-B* F | AGACACAGATCTCCAAGACCAACA |
| Human *HLA-B* R | CGTCGCAGCCGTACATCCT |
| Human *MYBBP1A* F | GACCGCTATGGCCTATTGAAG |
| Human *MYBBP1A* R | GGGCATATTTCATCTCGGACC |
| Human *CCL5* F | GAGTATTTCTACACCAGTGGCAAG |
| Human *CCL5* R | TCCCGAACCCATTTCTTCTCT |
| Human *IFIT2* F | AAGCACCTCAAAGGGCAAAAC |
| Human *IFIT2* R | TCGGCCCATGTGATAGTAGAC |
| Human *RSAD2* F | CAGCGTCAACTATCACTTCACT |
| Human *RSAD2* R | AACTCTACTTTGCAGAACCTCAC |
| Human *IFITM3* F | TCACACTGTCCAAACCTTCTTCT |
| Human *IFITM3* R | GCCCCCAGCACAGCCACCTCG |
| Mouse *Actb* F | GTGACGTTGACATCCGTAAAGA |
| Mouse *Actb* R | GCCGGACTCATCGTACTCC |
| Mouse *Il10* F | CTTACTGACTGGCATGAGGATCA |
| Mouse *Il10* R | GCAGCTCTAGGAGCATGTGG |
| Mouse *Il12a* F | CTGTGCCTTGGTAGCATCTATG |
| Mouse *Il12a* R | GCAGAGTCTCGCCATTATGATTC |
| Mouse *Il23a* F | ATGCTGGATTGCAGAGCAGTA |
| Mouse *Il23a* R | ACGGGGCACATTATTTTTAGTCT |
| Mouse *Tnf* F | CCTGTAGCCCACGTCGTAG |
| Mouse *Tnf* R | GGGAGTAGACAAGGTACAACCC |

**Supplementary Table 2** The detailed antibody information used in this study.

| **Antibodies** | **Manufacturer** | **Catalog number** |
| --- | --- | --- |
| **Antibodies for western blot** |  |  |
| Anti-STAT1 antibody | Cell Signaling Technology | 14994S |
| Anti-STAT1 (phospho S727) antibody | Abcam | ab109461 |
| Anti-IRF1 antibody | Cell Signaling Technology | 8478S |
| Anti-HLAB antibody | Abcam | ab225636 |
| Anti-NAT10 antibody | Abcam | ab194297 |
| Anti-p53 antibody | Proteintech | 60283-2-Ig |
| Anti-p53 antibody | Santa cruz | sc-126 |
| Anti-p53 antibody | Abcam | ab179477 |
| Anti-β-actin antibody | Cell Signaling Technology | 4970S |
| Anti-CXCL10 antibody | Proteintech | 10937-1-AP |
| Anti-CCL5 antibody | Proteintech | 12000-1-AP |
| Anti-IFIT2 antibody | Proteintech | 12604-1-AP |
| Anti-RSAD2 antibody | Proteintech | 28089-1-AP |
| Anti-IFITM3 antibody | Proteintech | 11714-1-AP |
| **Antibodies for immunohistochemistry** |  |  |
| Anti-CD3ε antibody | Cell Signaling Technology | 85061T |
| Anti-CD68 antibody | Cell Signaling Technology | 76437T |
| Anti-F4/80 antibody | Cell Signaling Technology | 70076T |
| Anti-Ki67 antibody | Abcam | ab15580 |
| Anti-STAT1 (phospho S727) antibody | Abcam | ab109461 |
| Anti-IRF1 antibody | Cell Signaling Technology | 8478S |
| Anti-HLAB antibody | Abcam | ab225636 |
| Anti-CD4 antibody | Cell Signaling Technology | 48274S |
| Anti-CD8 antibody | Cell Signaling Technology | 85336S |
| Anti-CXCL10 antibody | Proteintech | 10937-1-AP |
| Anti-CCL5 antibody | Abmart | TA5151 |
| Anti-IFIT2 antibody | Proteintech | 12604-1-AP |
| Anti-RSAD2 antibody | Proteintech | 28089-1-AP |
| Anti-IFITM3 antibody | Proteintech | 11714-1-AP |
| **Antibodies for immunoprecipitation** |  |  |
| Anti-p53 antibody | Proteintech | 10442-1-AP |
| Anti-MYBBP1A antibody | Proteintech | 14524-1-AP |
| Anti-N4-acetylcytidine (ac4C) antibody | Abcam | Ab252215 |
| Anti-NAT10 antibody | Abcam | ab194297 |
| **Antibodies for neutralization** |  |  |
| Anti-CXCL10 antibody | R&D Systems | MAB266 |
| Anti-CCL5 antibody | R&D Systems | MAB678 |
