# Supplementary material for: NAT10-mediated upregulation of GAS5 facilitates immune cell infiltration in non-small cell lung cancer via the MYBBP1A-p53/IRF1/type I interferon signaling axis
Source: Cell Death Discov. 2024 May 18;10:240. doi: 10.1038/s41420-024-01997-2 (PMC11102450; doi:10.1038/s41420-024-01997-2)

Fig. 2G


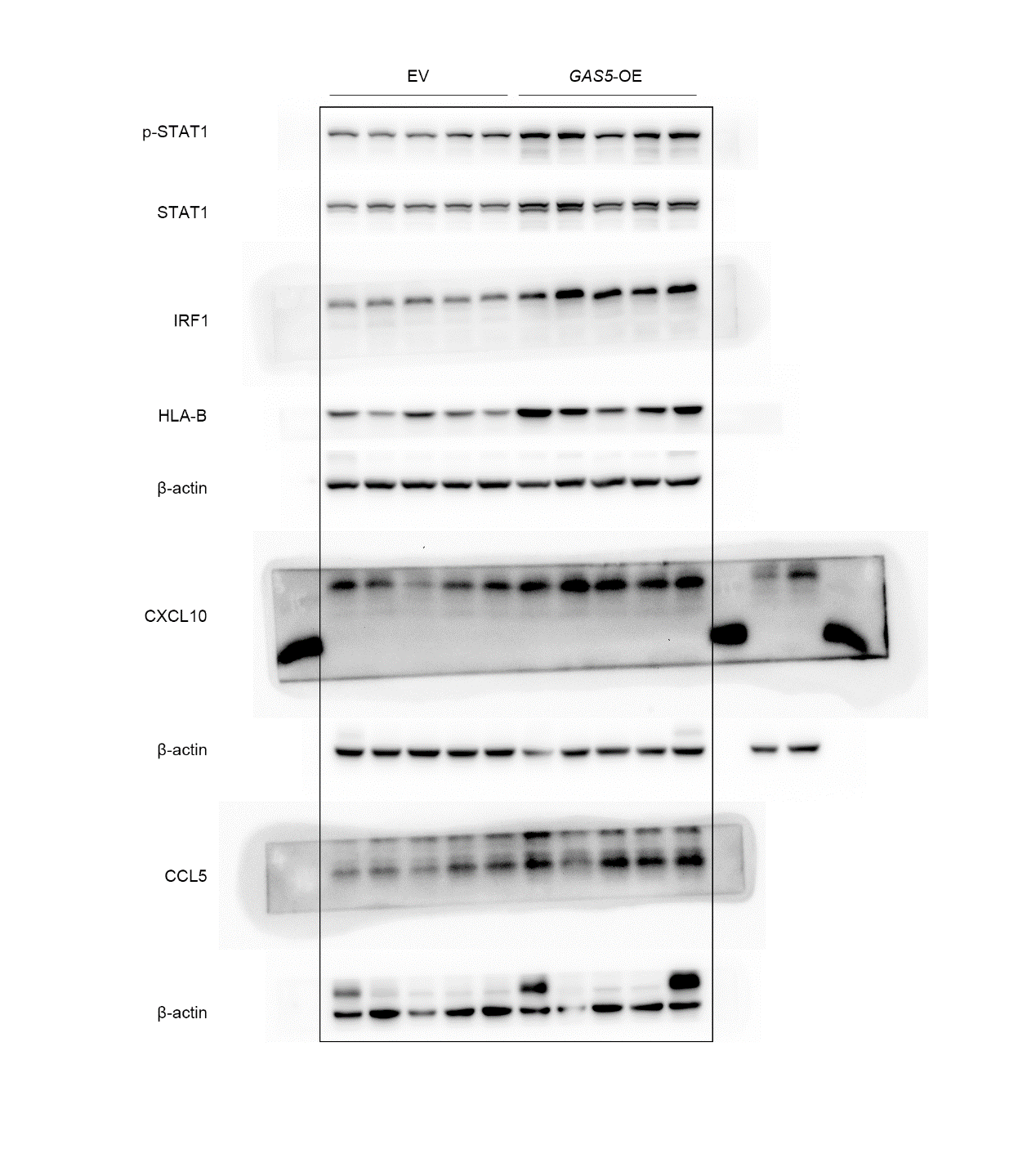


Fig. 3E


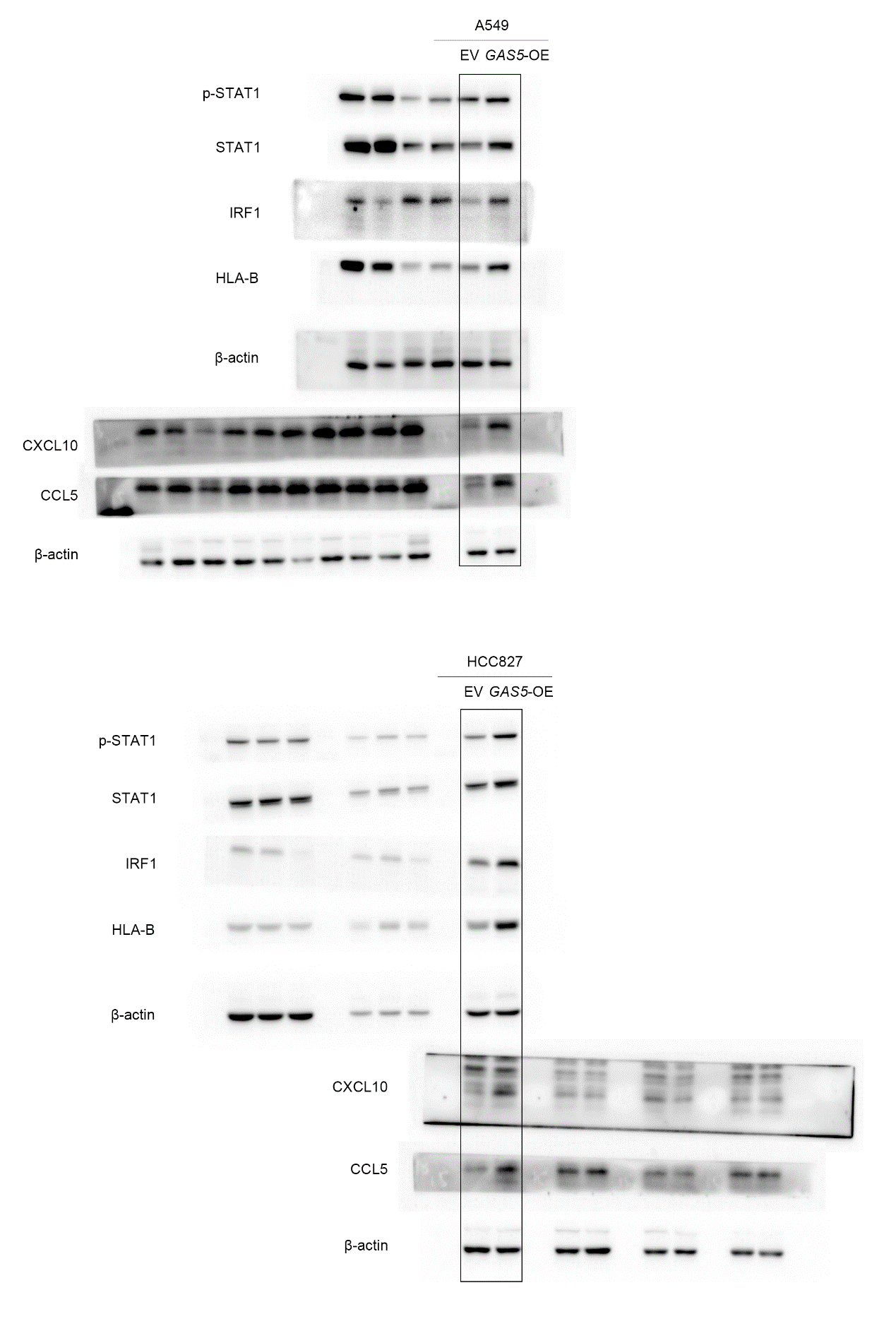


Fig. 3F


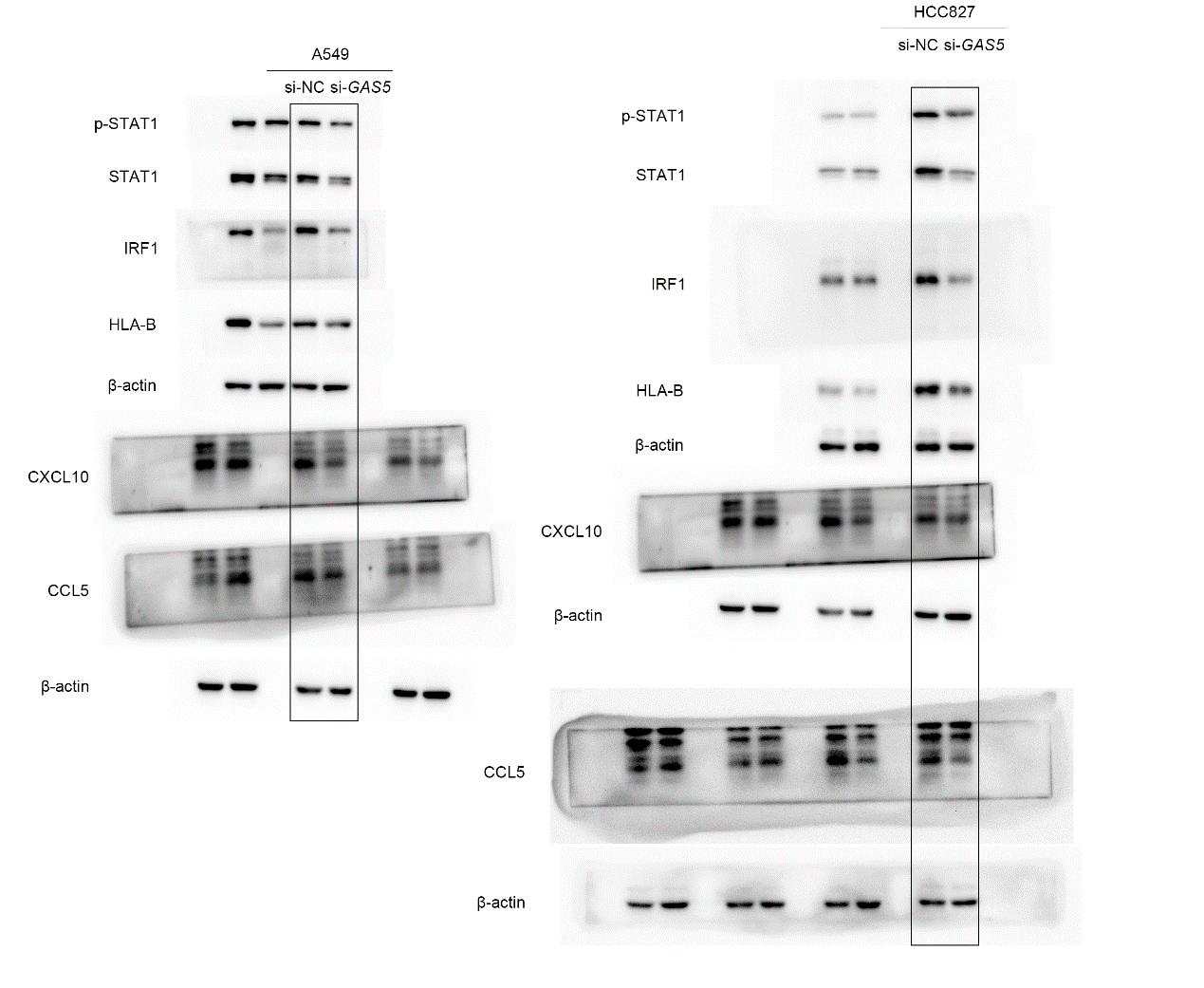


Fig. 4C


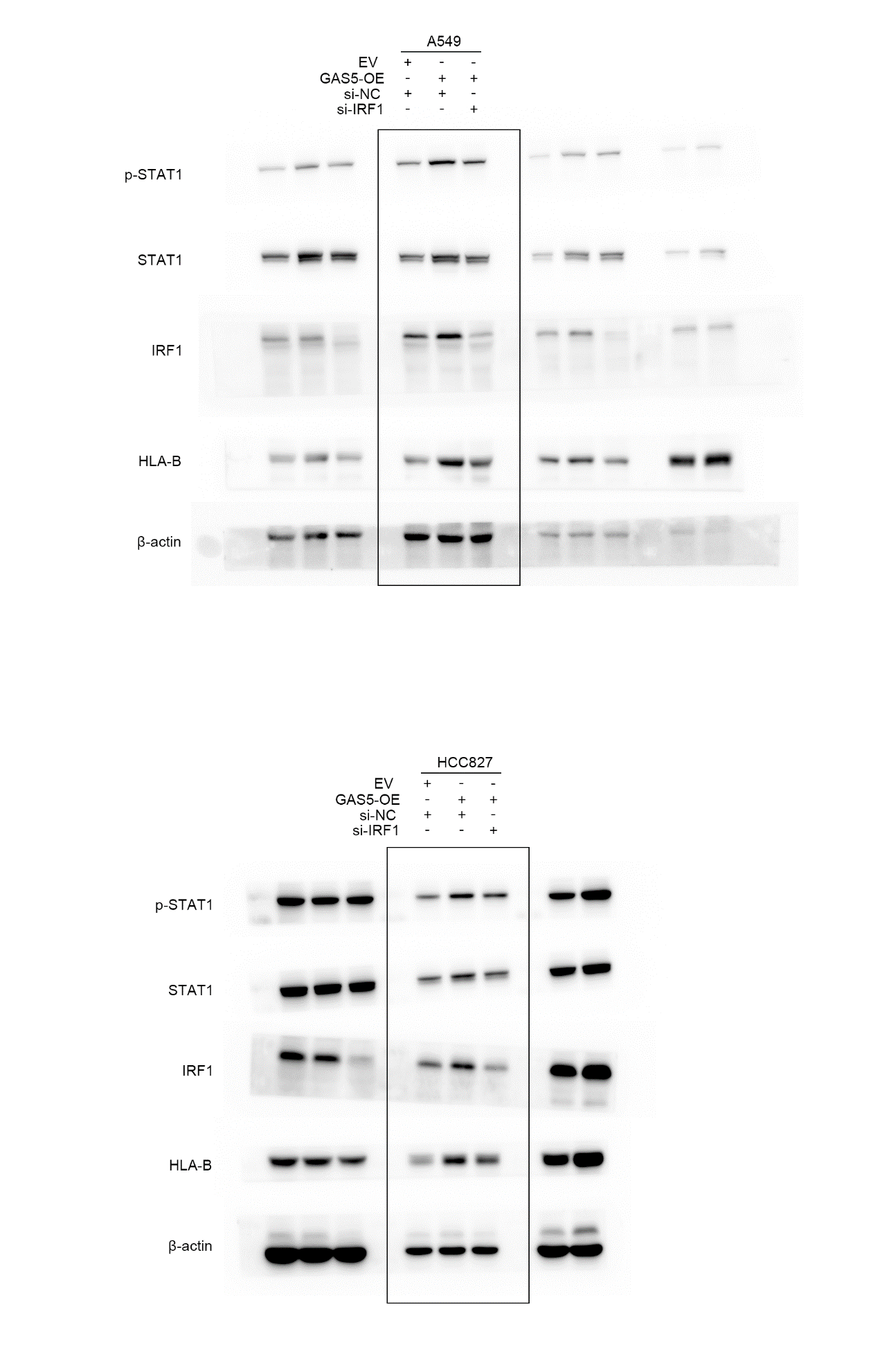


Fig. 4E


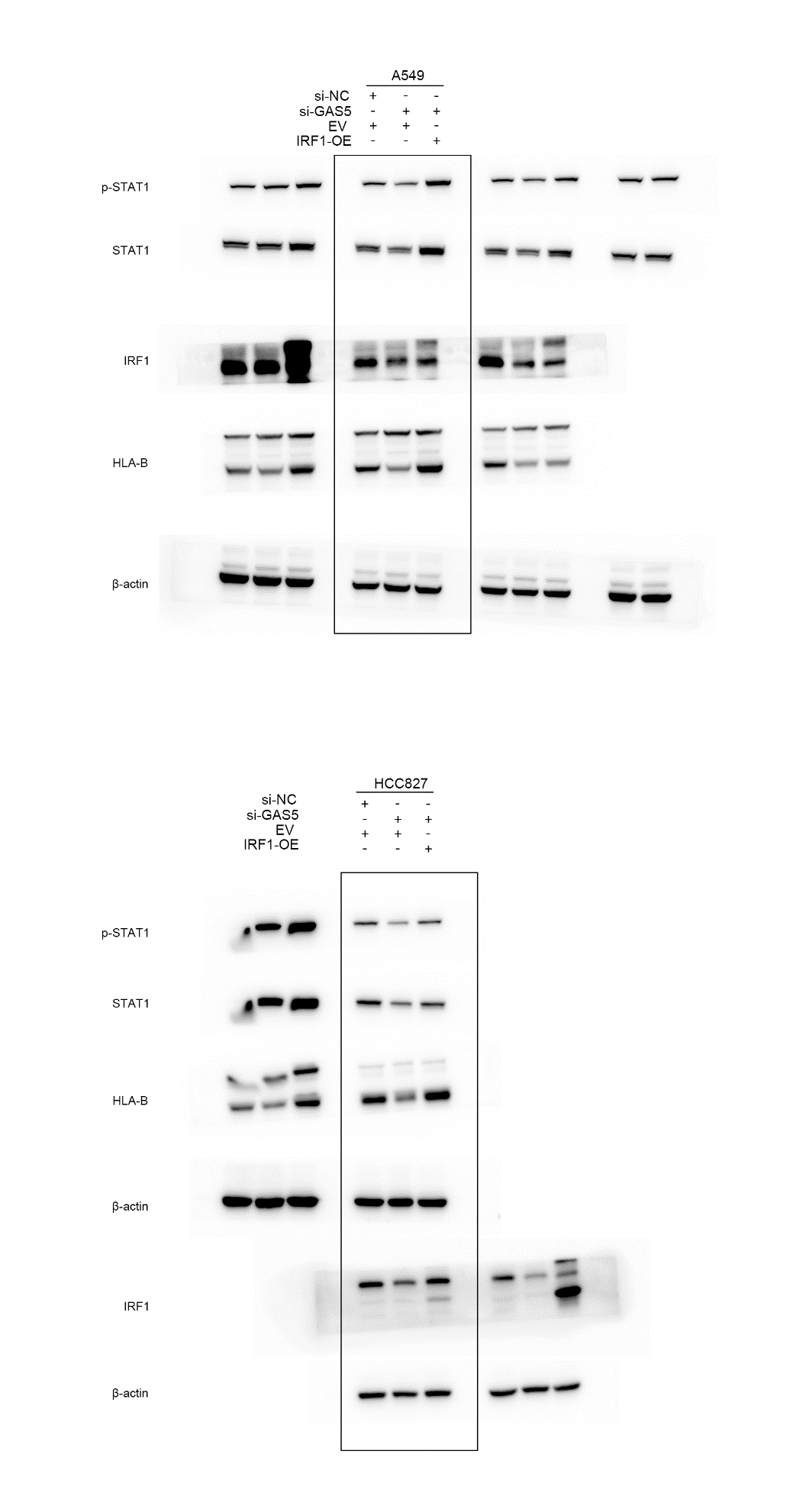


Fig. 5B


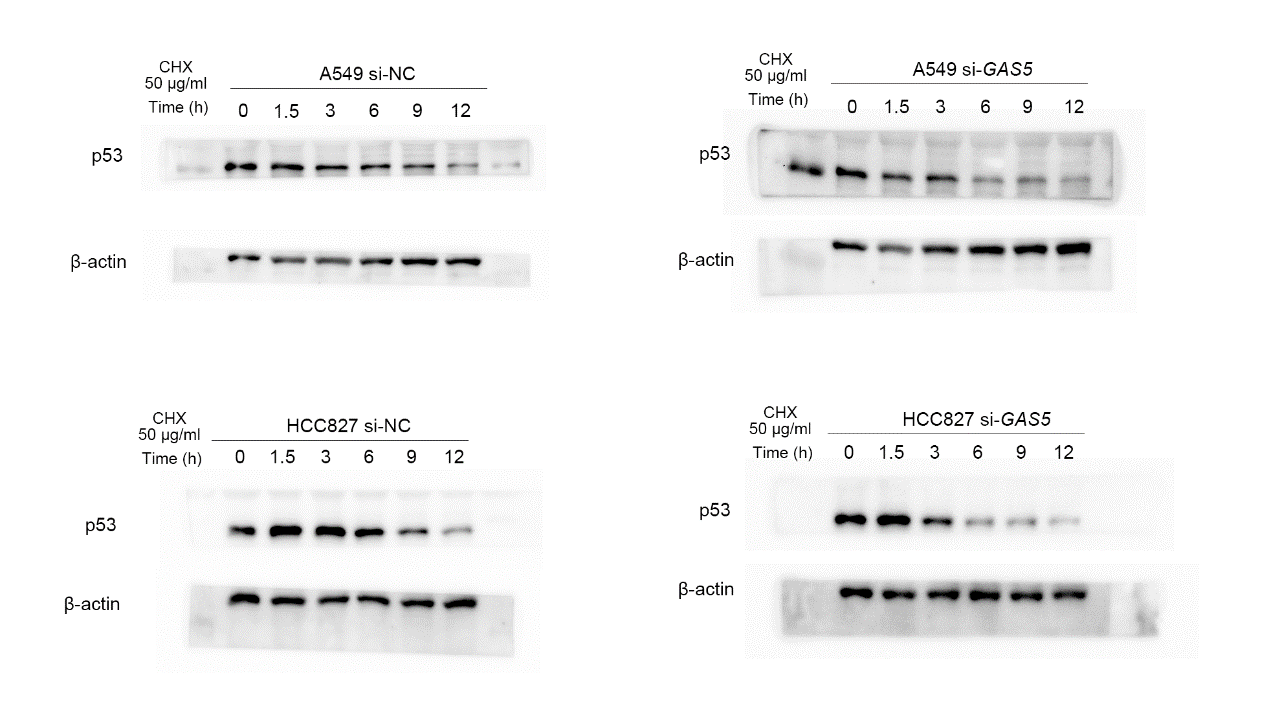


Fig. 5D


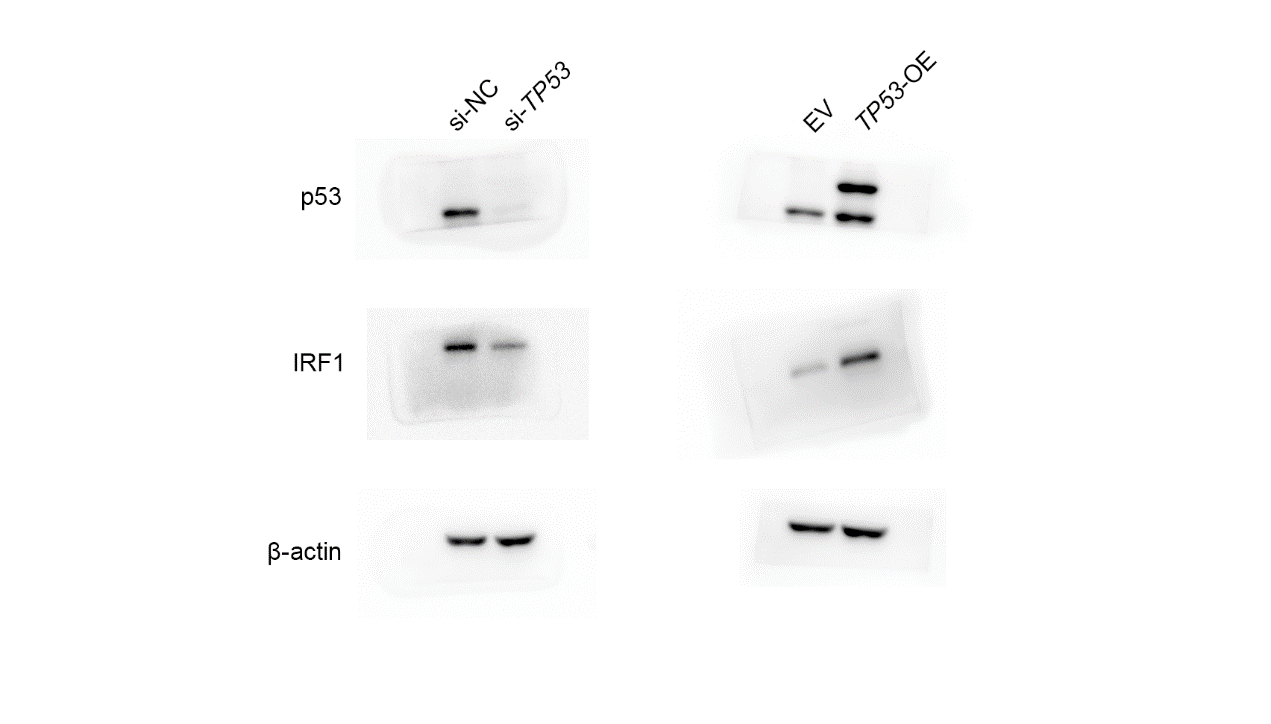


Fig. 5F


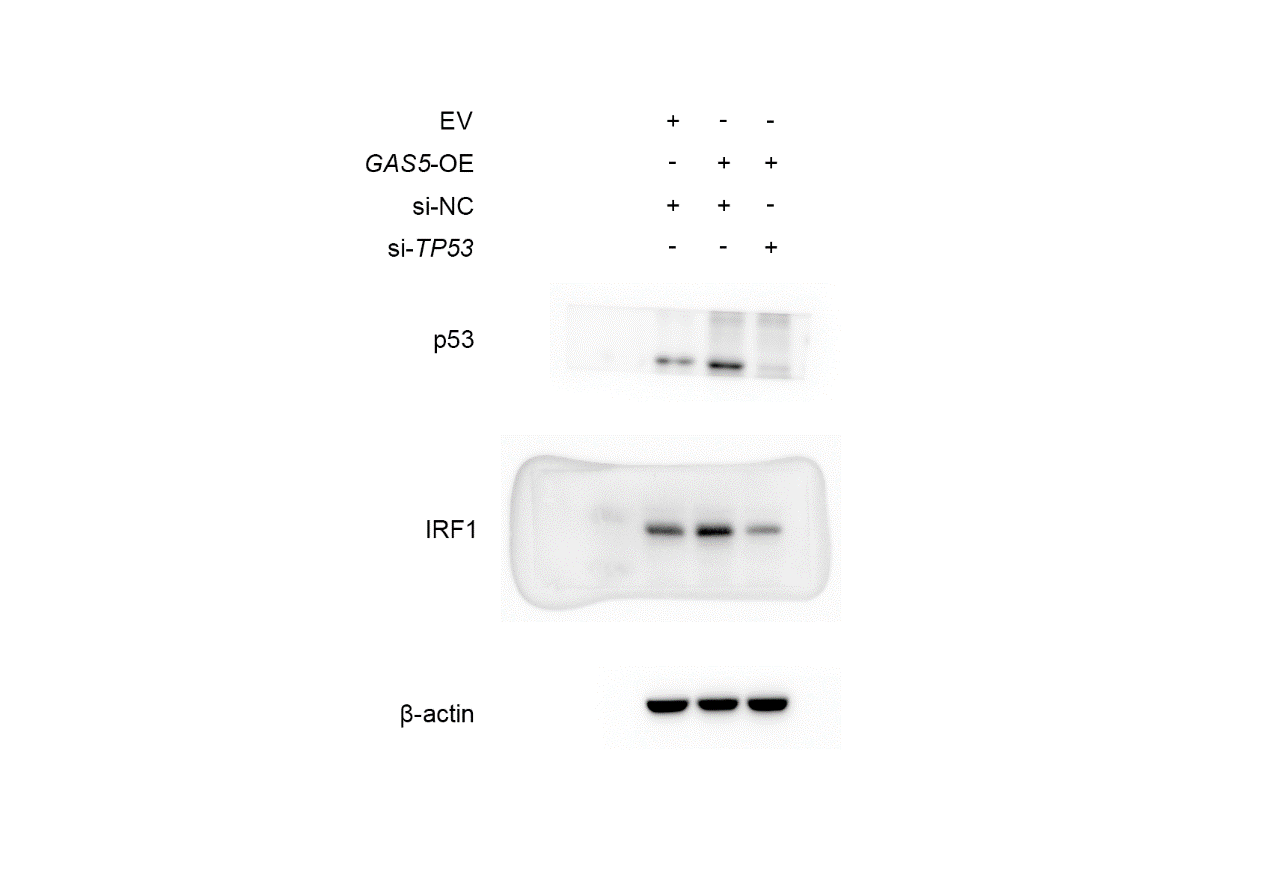


Fig. 5H


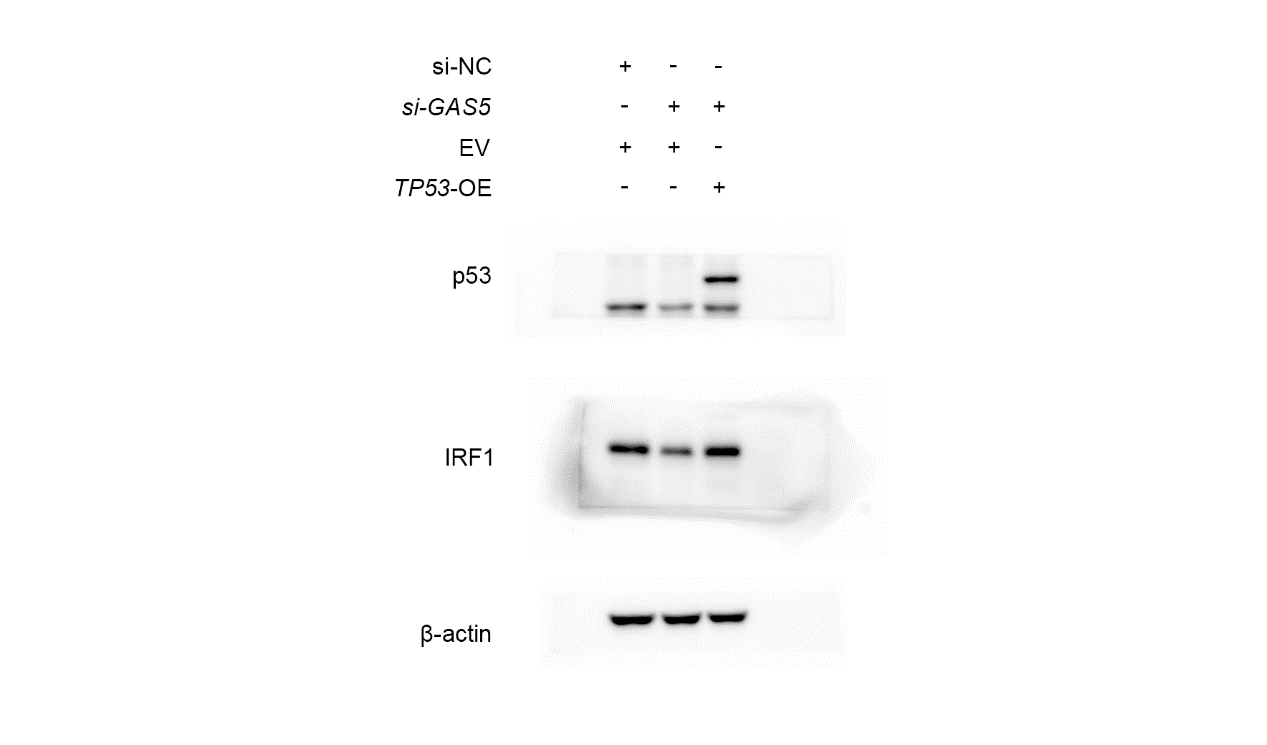


Fig. 6B


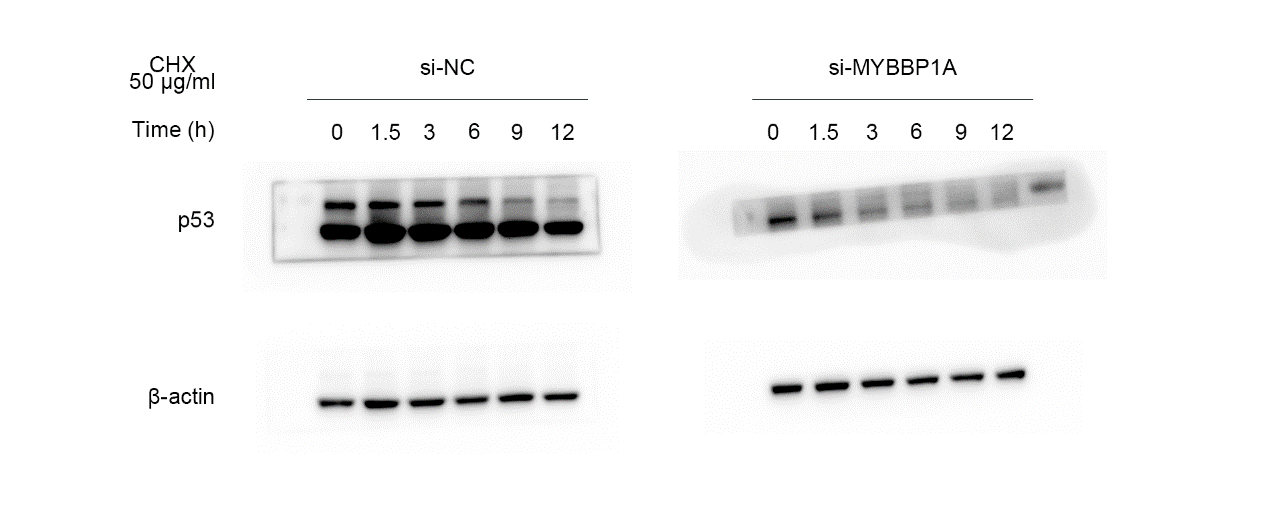


Fig. 6C


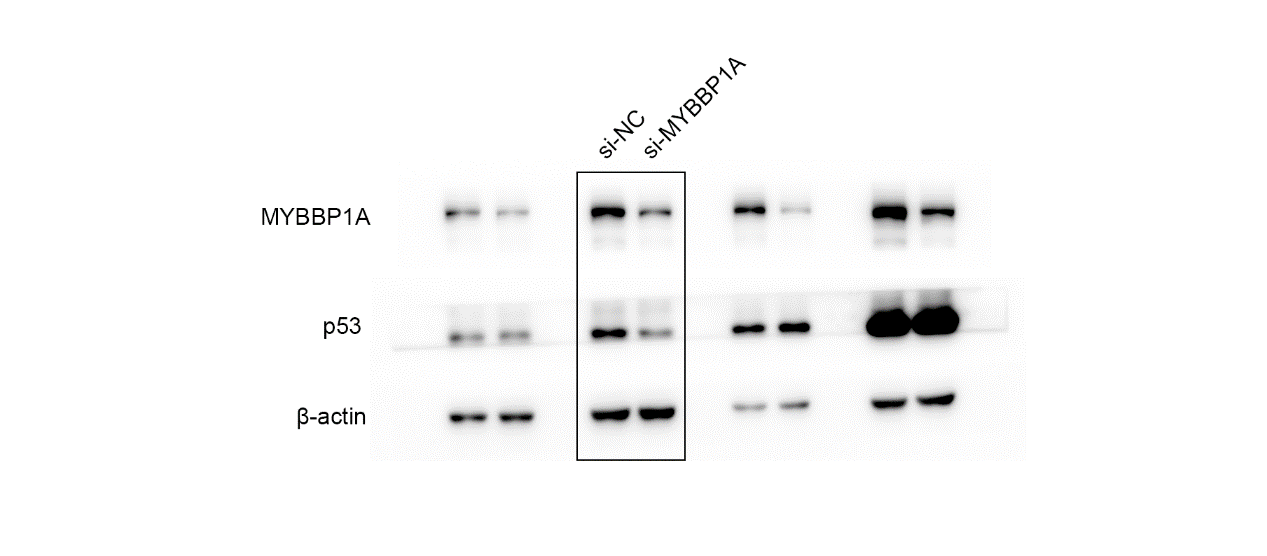


Fig. 6E


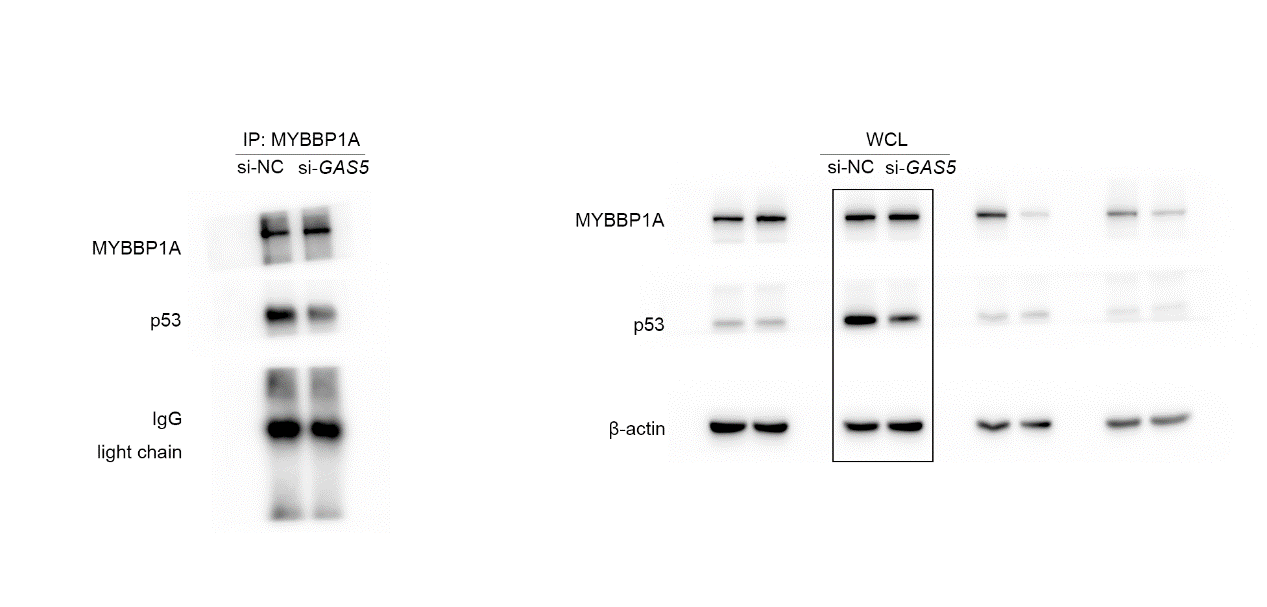


Fig. 6G


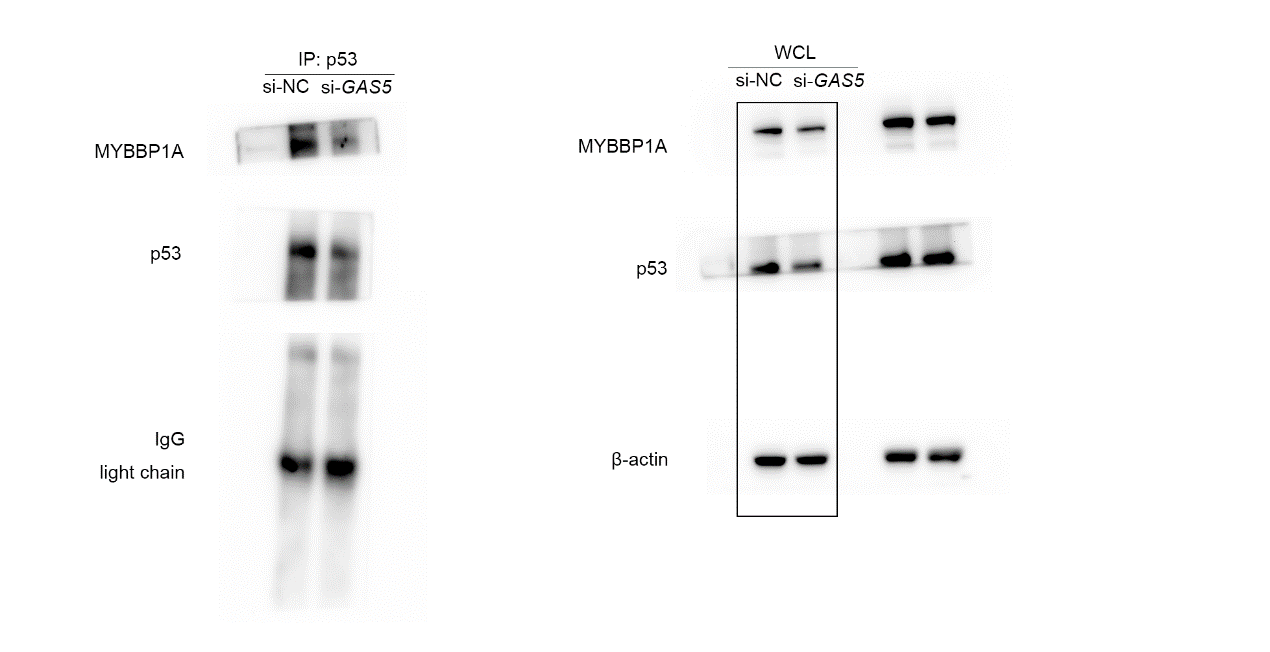


Fig. 7D


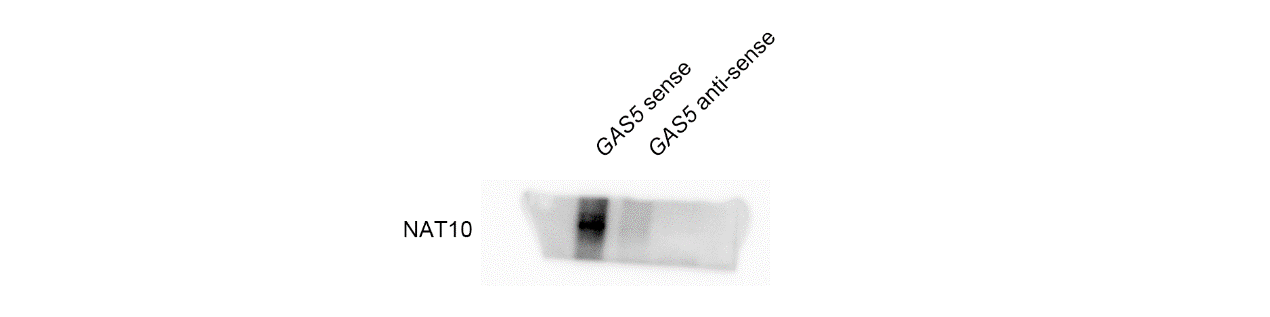


Supplementary fig. 1F


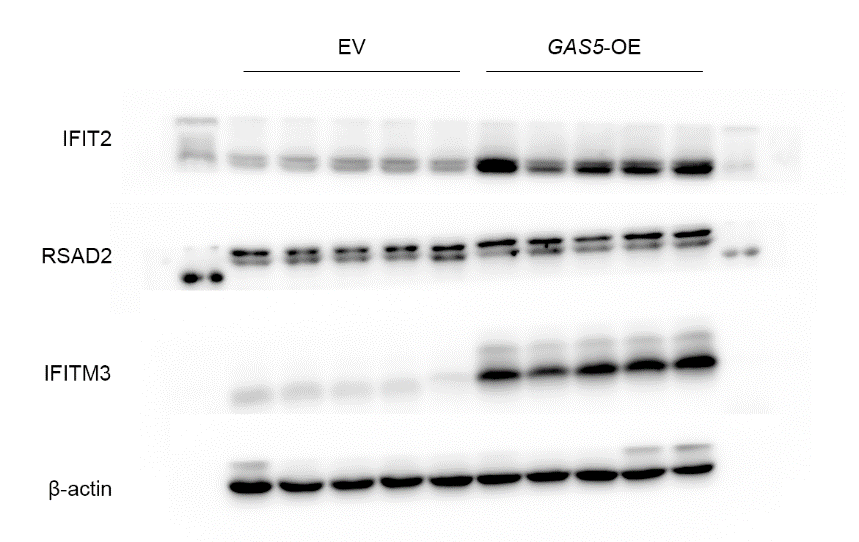


Supplementary fig. 2A


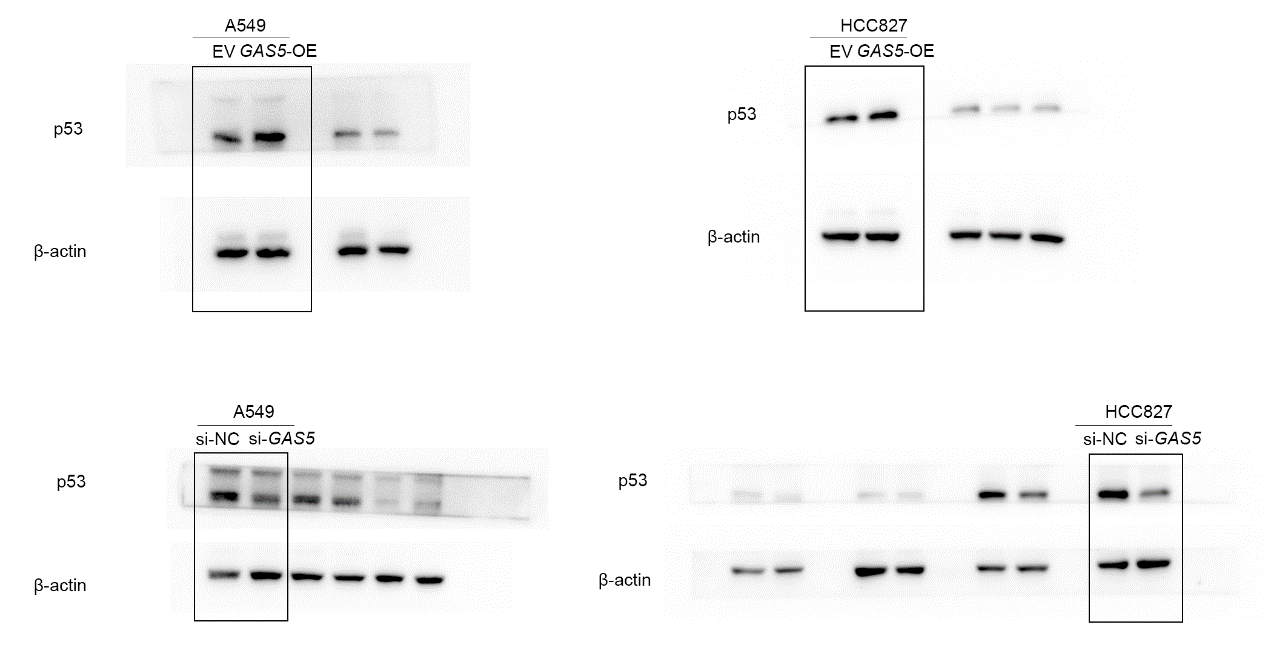


Supplementary fig. 2C


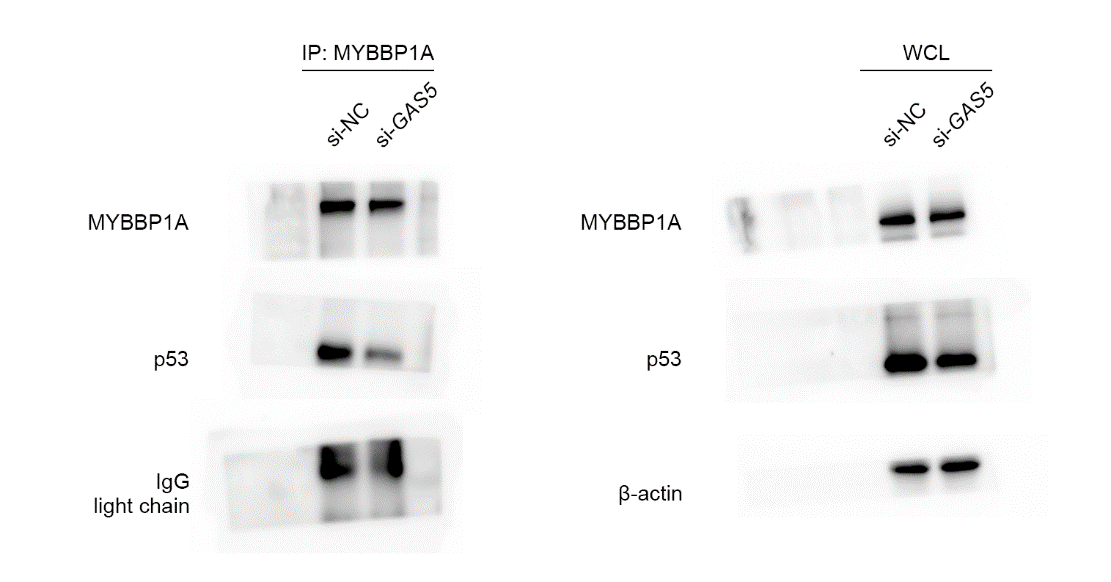


Supplementary fig. 2E


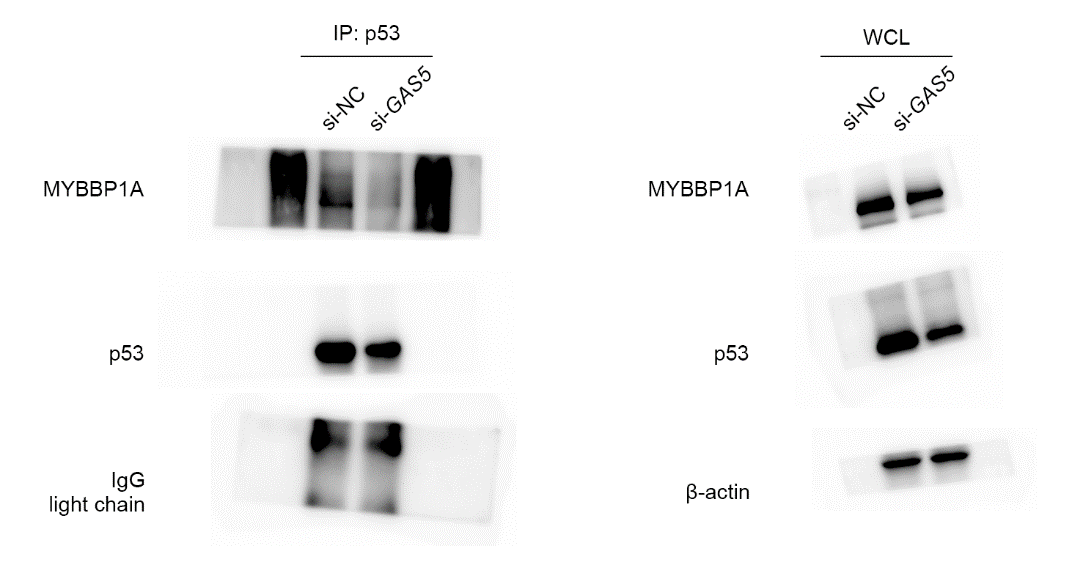

Supplement: Supplementary file 2 — Original western blots [file 41420_2024_1997_MOESM2_ESM.docx]
